# Unraveling Molecular Fingerprints of Catalytic Sulfur Poisoning at the Nanometer Scale with Near-Field Infrared Spectroscopy

**DOI:** 10.1021/jacs.2c03088

**Published:** 2022-04-29

**Authors:** Zafer Say, Melike Kaya, Çağıl Kaderoğlu, Yusuf Koçak, Kerem Emre Ercan, Abel Tetteh Sika-Nartey, Ahsan Jalal, Ahmet Arda Turk, Christoph Langhammer, Mirali Jahangirzadeh Varjovi, Engin Durgun, Emrah Ozensoy

**Affiliations:** † Department of Chemistry, Bilkent University, 06800 Ankara, Turkey; ‡ Department of Materials Science and Nanotechnology Engineering, TOBB University of Economics and Technology, 06510 Ankara, Turkey; § Department of Physics, Chalmers University of Technology, SE-412-96 Gothenburg, Sweden; ∥ Institute of Acceleration Technologies, Ankara University, 06830 Ankara, Turkey; ⊥ Turkish Accelerator and Radiation Laboratory (TARLA), 06830 Ankara, Turkey; # Department of Physics Engineering, Ankara University, 06100 Ankara, Turkey; ∇ UNAM—National Nanotechnology Research Center, Bilkent University, 06800 Bilkent, Ankara, Turkey

## Abstract

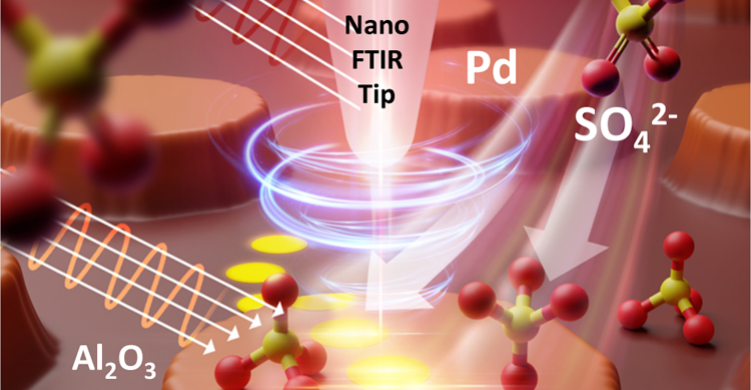

Fundamental understanding of catalytic deactivation phenomena such as sulfur poisoning occurring on metal/metal-oxide interfaces is essential for the development of high-performance heterogeneous catalysts with extended lifetimes. Unambiguous identification of catalytic poisoning species requires experimental methods simultaneously delivering accurate information regarding adsorption sites and adsorption geometries of adsorbates with nanometer-scale spatial resolution, as well as their detailed chemical structure and surface functional groups. However, to date, it has not been possible to study catalytic sulfur poisoning of metal/metal-oxide interfaces at the nanometer scale without sacrificing chemical definition. Here, we demonstrate that near-field nano-infrared spectroscopy can effectively identify the chemical nature, adsorption sites, and adsorption geometries of sulfur-based catalytic poisons on a Pd(nanodisk)/Al_2_O_3_ (thin-film) planar model catalyst surface at the nanometer scale. The current results reveal striking variations in the nature of sulfate species from one nanoparticle to another, vast alterations of sulfur poisoning on a single Pd nanoparticle as well as at the assortment of sulfate species at the active metal–metal-oxide support interfacial sites. These findings provide critical molecular-level insights crucial for the development of long-lifetime precious metal catalysts resistant toward deactivation by sulfur.

## Introduction

Since the very first introduction of the concept of catalysis by the Scottish chemist Elizabeth Fulhame in 1794,^1^ catalysis has been a major industrial driving force with a global market that is expected to reach up to $34 billion by 2025, with an annual growth rate of 4.5%.^2^ Hence, improvement of catalyst lifetime and durability continues to be a critical field of interdisciplinary research. As Paul Sabatier proposed in 1913,^3^ reactants should bind to a heterogeneous catalyst surface with sufficient strength but not too strongly so that they do not cause catalyst deactivation. Many catalytic processes utilize platinum group metal (PGM) nanoparticles (NPs), such as Pd, Pt, Rh, Ir, Ru, Os, and their multimetallic forms with complex sizes, shapes, geometries, and composition, which are dispersed on porous metal-oxide support materials (e.g., Al_2_O_3_, SiO_2_, TiO_2_, zeolites). Sulfur-containing species, such as SO*_x_*, may bind to PGM NPs as well as to the metal-oxide support material in a strong and often irreversible manner resulting in catalytic deactivation, which shortens catalyst lifetime and decreases catalytic conversion and selectivity. Catalytic deactivation due to sulfur poisoning is a widely encountered critical problem in numerous industrial chemical processes, including catalytic exhaust emission control technologies,^4,5^ catalytic hydrocarbon combustion systems,^6^ solid oxide fuel cells (SOFC),^7^ photo/electrocatalytic water splitting,^8^ mass production of sulfuric acid,^9^ and the industrial (modified) Claus process for elemental sulfur production.^10^ Its molecular-level mechanistic understanding is, however, to date, not fully available. This is because a comprehensive understanding of sulfur poisoning requires experimental methods that enable nanometer-scale spatial resolution without sacrificing information about chemical bonding, functional groups, adsorption sites, and adsorption geometries.

Unfortunately, most of the existing conventional spectroscopic, microscopic, and diffraction techniques utilized for the characterization of catalytic metal/metal-oxide interfaces suffer from a trade-off between spatial resolution and chemical structure/bonding definition.^11,12^ For instance, techniques providing very high spatial resolution, such as scanning tunneling/atomic force/transmission electron microscopy (STM/AFM/TEM), energy-dispersive X-ray analysis (EDX), or electron energy loss spectroscopy (EELS), typically cannot provide unambiguous information about chemical functional groups, molecular structure, and adsorption geometries of catalytic adsorbates at the same time.^11,12^ Such techniques are also frequently limited to unrealistically low pressures (<10^–12^ atm) and cryogenic temperatures (<20 K) for molecular-level data acquisition.^13,14^ Furthermore, some of these techniques may also lead to sample damage due to the utilization of energetic electrons or photons with high flux. On the other hand, optical far-field spectroscopy/microscopy methods involving infrared (IR) photons yield detailed chemical/bonding/adsorption geometry information without sample damage. However, they are limited to a theoretical spatial resolution of >1.2 μm (for mid-IR investigations using photon wavelengths within 2.5–20.0 μm), due to the diffraction limit proposed by Ernst Abbe in 1873,^15^ giving rise to ensemble averaging of thousands/millions of nonuniform catalytic nanoparticles. Thus, conventional far-field IR spectroscopic/microscopic studies on metal/metal-oxide catalytic interfaces lead to convoluted signals originating from multiple domains, which render distinct identification of particular adsorbates on different domains unattainable. Plasmonic near-field nanoimaging^16^ and tip-enhanced Raman spectroscopy (TERS)^17,18^ are other potent high-spatial-resolution techniques, where the latter may provide signals from the direct vicinity of an AFM tip, with a spatial resolution of <20 nm. However, none of these high-resolution techniques have been applied so far to study the deactivation of supported PGM nanoparticles by sulfur under relevant temperatures and pressures, leaving the following key questions unanswered: (i) Where exactly do the inhibitors/poisons bind on the catalyst surface, e.g., on PGM active sites and/or on the support material? (ii) What kind of functional groups do the adsorbed poisoning species possess? (iii) What are the adsorption geometries of such poisons on the catalyst surface?

**Figure 1:**
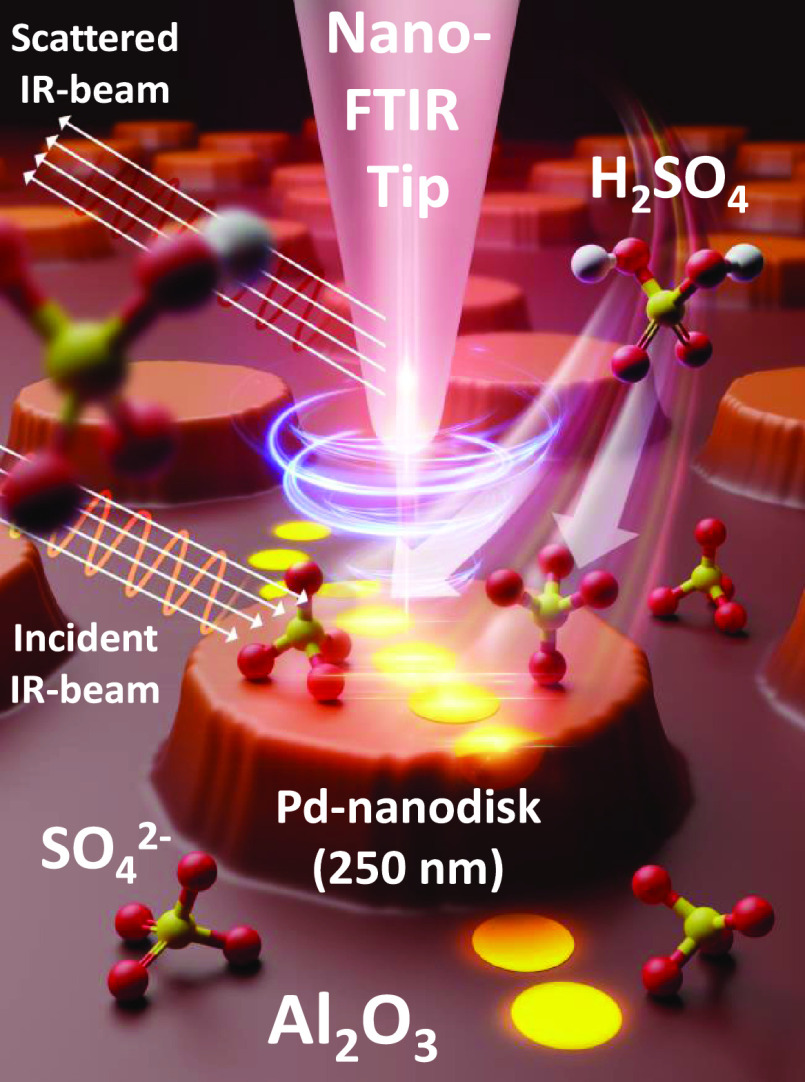
Artist’s (not drawn to scale) rendition of the ex situ nano-FTIR spectroscopic monitoring of the sulfur-poisoning species, i.e., different types of sulfates, introduced on the nanofabricated 2D-Pd(nanodisk)/Al_2_O_3_ planar model catalyst surface via H_2_SO_4_ exposure.

Here, we employ scattering-type scanning near-field optical microscopy (s-SNOM)^19−29^-based near-field nano-Fourier transform infrared spectroscopy (nano-FTIR) (see Supporting Information, SI Section 1 for more details) to show that the adsorption site, functional group (i.e., internal bonding configuration), and adsorption geometries of sulfur-poisoning species can be distinguished and chemically identified at the nanometer scale relevant to catalysis. For this purpose, we use a two-dimensional (2D) model catalyst surface comprised of an array of nanofabricated shape-defined Pd nanodisks on a planar Al_2_O_3_ thin-film support grown on an oxidized Si(100) substrate. This sample is treated with 1.0 × 10^–2^ M H_2_SO_4_(aq) at 383 K in air to introduce sulfates on the catalytic metal/metal-oxide (Pd/Al_2_O_3_) interface as poisoning species (Figure 1). As a key result, we show that Pd nanodisks are poisoned with sulfates, which are predominantly adsorbed with a 3-fold adsorption geometry on Pd^0^ (metallic) sites of Pd(111) surface facets, along with a smaller contribution from sulfates adsorbed with 3-fold or 1-fold geometry (i.e., with a *C*_3*v*_ symmetry) on electron-deficient Pd^*x*+^ sites. On the other hand, sulfur-poisoning species on the Al_2_O_3_ thin-film support revealed notable differences in their nano-FTIR vibrational signatures, indicating the presence of sulfates adsorbed with 2-fold (bidentate) configuration, as well as bulk-like sulfates (i.e., Al_2_(SO_4_)_3_). Furthermore, our results illustrate that the types of adsorbed sulfate species can be modified by altering the extent of poisoning and by changing the Pd nanodisk surface morphology as well as the oxidation states of the Pd adsorption sites at the nanometer scale. Accordingly, we demonstrate that the detection of the significant variations in the adsorption characteristics of sulfate species (a) on different Pd nanoparticles, (b) on a single Pd nanoparticle, and (c) at the active metal (Pd)/metal-oxide support (Al_2_O_3_) interface could be possible via the nano-FTIR technique. By means of subsequent regeneration experiments, we also establish that most of the sulfur-poisoning species can be removed both from the mildly poisoned Pd nanodisks and the Al_2_O_3_ support surface of the 2D model catalyst via catalytic reduction with H_2_ at 573 K.

## Results and Discussion

### Model Catalyst Nanofabrication

**Figure 2:**
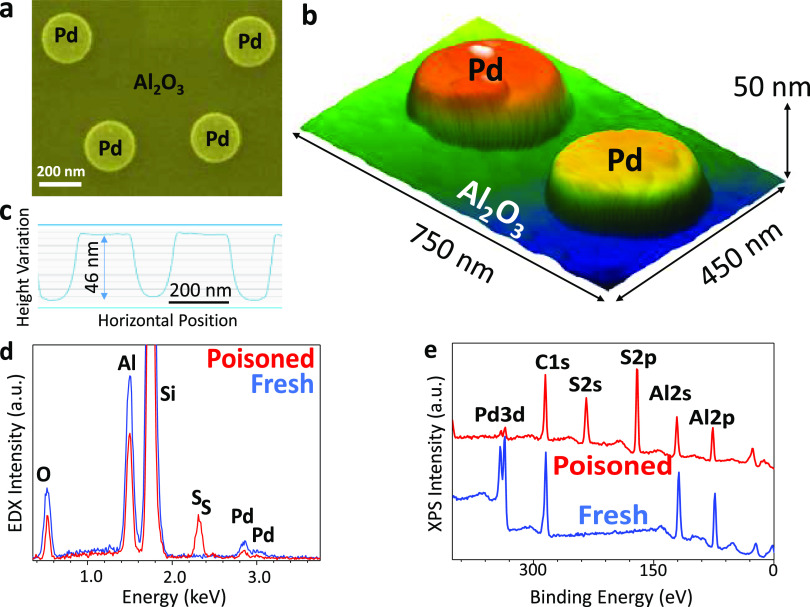
(a) Scanning electron microscopy (SEM) image, (b) (false color) total IR-reflection s-SNOM image, and (c) AFM height variation profile of the freshly prepared 2D-Pd(nanodisk)/Al_2_O_3_ model catalyst surface. (d) EDX and (e) XPS survey spectra of the fresh (blue) and sulfur-poisoned (red) model catalyst.

The 2D-Pd(nanodisk)/Al_2_O_3_ planar model catalyst was fabricated using the hole-mask colloidal lithography nanofabrication method,^30^ which enables the production of quasi-random arrays of shape-defined Pd nanodisks deposited on a planar Al_2_O_3_ thin film with a thickness of ca. 300 nm, grown with sputter deposition on a thermally oxidized Si(100) substrate (for further details on model catalyst nanofabrication, see Figure S1 and SI Section 2). The Pd nanodisks had an average diameter of 250 ± 20 nm (Figure 2a,b) and an average height of 50 ± 5 nm (Figure 2b,c). The chemical composition of the freshly prepared Pd/Al_2_O_3_ model catalyst surface was also characterized with energy-dispersive X-ray analysis (EDX) (Figure 2d) and X-ray photoelectron spectroscopy (XPS, Figures 2e and S2a–f), verifying the presence of Pd, Al, O, and Si. The corresponding measurements on a H_2_SO_4_-exposed (i.e., mildly poisoned) sample also yielded additional strong S signals, confirming the sulfur-poisoning effect. Furthermore, detailed XPS analysis of the Pd 3d region of the fresh and mildly sulfur-poisoned model catalyst surfaces indicated that the relative abundance of Pd^0^ to Pd^2+^ sites was 3.2 and 1.9, respectively (SI Section 3 and Figure S2a,b). In other words, fresh Pd nanodisks exhibited a mostly metallic nature with a notable contribution from Pd^2+^ species, while the relative abundance of the Pd^2+^ species increased with sulfur poisoning.

### Distinguishing the Adsorbates Formed on Pd Nanodisks and the Al_2_O_3_ Support with Nanometer-Scale Resolution upon Catalytic Deactivation by Sulfur

**Figure 3:**
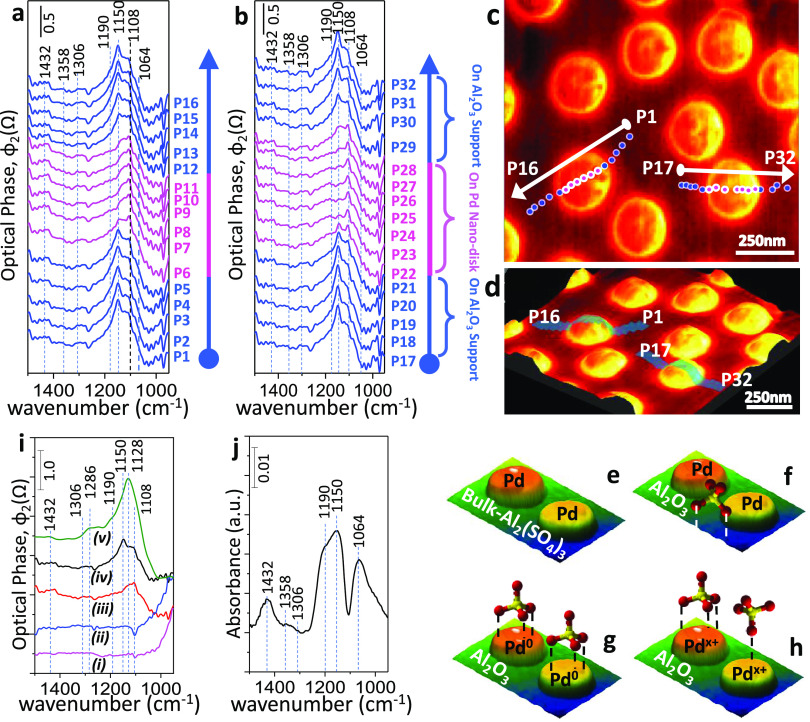
(a, b) Nano-FTIR spectra collected from the line scans across two independent mildly sulfur-poisoned single Pd nanodisks on Al_2_O_3_. Nano-FTIR spectra labeled with blue color were acquired directly from the Al_2_O_3_ support sites (corresponding to points P1–P5, P12–P16, P17–P21, and P29–P32 in panel (c)), while the purple spectra were acquired directly from the Pd nanodisks (corresponding to points P6–P11 and P22–P28 in panel (d)). (c) 2D and (d) 3D representations of total IR-reflection s-SNOM images of the mildly sulfur-poisoned Pd(nanodisk)/Al_2_O_3_ (thin-film) planar model catalyst surface showing the locations of the points where the nano-FTIR line scan spectra were acquired. (e–h) Schematic (not drawn to scale) illustration of the prominent sulfate species detected in the nano-FTIR spectra given in panels (a, b). (i) Nano-FTIR spectra obtained from: pristine Pd nanodisk (*i*) and pristine Al_2_O_3_ (*ii*) domains of the clean Pd nanodisk/Al_2_O_3_ (thin-film)/Si(100) surface, mildly sulfur-poisoned Pd nanodisk (*iii*), and mildly sulfur-poisoned Al_2_O_3_ domains (*iv*) of Pd nanodisk/Al_2_O_3_ (thin-film)/Si(100) surface, as well as the mildly sulfur-poisoned Al_2_O_3_ (thin-film)/Si(100) surface that does not have any Pd disks (v). For spectra *i*, *ii*, and *v*, the nano-FTIR spectrum of the Si wafer was used as the background. Spectra *i* and *ii* were used as the background for spectra *iii* and *iv*, respectively. (j) Far-field ATR-IR spectrum of the identical mildly sulfur-poisoned Pd(nanodisk)/Al_2_O_3_ model catalyst given in panels (a–i).

We utilized s-SNOM-based nano-FTIR spectroscopy^19,27^ to identify and differentiate the adsorbates generated upon sulfur poisoning of the Pd(nanodisk)/Al_2_O_3_ model catalyst surface (Figure 3a,b). Pd nanodisks were readily discerned in s-SNOM imaging (Figure 3c,d) due to their distinct geometry and topography, eliminating the need for additional elemental characterization for differentiating PGM active sites from the catalytic support domains. To mimic some of the relevant adsorbates produced during a typical catalytic sulfur-poisoning event, we exposed the pristine model catalyst surface to 1.0 × 10^–2^ M H_2_SO_4_(aq). Note that the concentration of H_2_SO_4_(aq) used in the current catalytic deactivation protocol (see Methods section for details) was optimized via control experiments. We observed that higher concentrations of H_2_SO_4_(aq) (e.g., 1 × 10^–1^ or 1.0 M) or longer exposure durations led to the leaching of Pd from the Pd nanodisks and distortion of the Pd nanodisk geometry, while lower concentrations (e.g., 1.0 × 10^–3^ or 1.0 × 10^–4^ M) of H_2_SO_4_(aq) resulted in low signal-to-noise ratio (S/N) in nano-FTIR spectra. In the next step, sulfuric-acid-treated model catalysts were annealed at 383 K in air to desorb the solvent and remove weakly bound physisorbed/chemisorbed species (e.g., molecular water). This particular sulfur-poisoning protocol was designed to: (i) ensure the generation of strongly bound sulfate species, which play a leading role in catalytic deactivation of PGM by sulfur, (ii) remove the mobile or weakly bound adsorbates, which otherwise can be transported or swept off by the tip during nano-FTIR spectroscopic measurements, thereby decreasing the data reproducibility and s-SNOM image quality, (iii) avoid the potential ambiguities in the nano-FTIR spectroscopic data analysis by eliminating adsorbates with severely overlapping vibrational spectroscopic features, such as SO_2_, SO_3_, SO_3_^2–^, HSO_3_^–^, HSO_4_^–^, and H_3_O^+^–SO_4_^2–^ (hydronium sulfate), which typically appear in the earlier stages of catalytic sulfur poisoning and eventually transform into sulfates upon extensive poisoning.^31−41^

Adsorption of sulfuric acid anions (i.e., HSO_4_^–^(ads) and SO_4_^2–^(ads)) has been extensively studied via FTIR, diffuse reflectance FTIR (DRIFTS), surface-sensitive infrared reflection absorption spectroscopy (IRAS), and sum frequency generation (SFG) on different precious metal surfaces such as Au,^37^ Rh,^32^ Pt,^39,41,42^ and Pd.^34,35^ H_2_SO_4_ adsorption was most comprehensively investigated on Pd and Pt single-crystal planar model catalysts in aqueous electrochemical systems via IRAS, SFG and also with density functional theory (DFT) modeling.^43,44^ Until recently, there has been a long-standing debate about whether HSO_4_^–^ and/or SO_4_^2–^ are adsorbed on Pt surfaces upon H_2_SO_4_(aq) exposure. As discussed comprehensively in SI Section 4 and Figure S3, recent studies^34−42,45−56^ showed conclusively that only sulfate species existed on various model catalyst surfaces upon H_2_SO_4_(aq) adsorption. Additional discussion on the fact that sulfates are the most prominent adsorbed species generated upon H_2_SO_4_(aq) adsorption on Pd(nanodisk)/Al_2_O_3_ planar model catalyst surfaces is given in SI Section 4.

During the nano-FTIR experiments presented in Figure 3a,b, we acquired spectra from a series of points aligned across two different sulfur-poisoned Pd nanodisks (Figure 3c,d). In such nano-FTIR spectroscopic line scans, we first started to register nano-FTIR spectra on the Al_2_O_3_ region, then continued across the Pd nanodisk, and finally ended up on the Al_2_O_3_ domains on the opposite side of the Pd nanodisk. Figure 3a,b, depicts two of these independent nano-FTIR line scans obtained across two separate Pd nanodisks. These independent measurements clearly demonstrate the reproducibility of the nano-FTIR signals and verify that these signals are among some of the valid representations of the overall model catalyst surface. Furthermore, they illustrate notable differences (i.e., spectral shifts and variations in line shapes) between the Al_2_O_3_ support (blue spectra) and the Pd nanodisks (purple spectra). This important observation suggests that sulfur-poisoning species on single Pd nanodisks can be differentiated from those of Al_2_O_3_ support sites with high spatial resolution at the nanometer scale.

The strength of the near-field signal can vary as a function of the tip surface interaction, tip surface distance, tip/disk geometry, and the near-field coupling between neighboring disks.^57,58^ Due to the relatively large distance between the Pd nanodisks used in the current work, near-field coupling between neighboring Pd disks seems unlikely.^57,59^ Furthermore, localized surface plasmon resonance (LSPR) effects for the currently utilized 250 nm diameter Pd nanodisks are expected to be in near-IR wavelengths (ca. 850 nm), while the currently used excitation source in nano-FTIR experiments operates in mid-IR wavelengths (6060–11 904 nm). On the other hand, minor asymmetry in the brightness of s-SNOM images of Pd disks given in Figure 3c has some resemblance to that of a former study by García-Etxarri et al.^57^ on 100 nm wide Au nanodisks utilizing a metallic SNOM tip but excited with 633 nm (visible) irradiation. In this former work, the aforementioned observation was attributed to the modification of the near-field amplitude over Au disks by the s-SNOM tip yielding dark regions close to the incoming radiation and brighter regions farther away from the incoming radiation. Thus, only some of the minor nano-FTIR spectral variations observed for the Pd disks given in Figure 3a,b of the current work can be attributed to such antenna effects. In other words, it is likely that contribution from such effects to the nano-FTIR spectral features is likely to be very weak (due to the fact that the current experiments utilize a mid-IR source with wavelengths much higher than the near-IR resonance wavelengths of the 250 nm Pd disks and also the Pd nanodisks have ca. 50% smaller LSPR extinction cross sections than that of Au disks).^60^ This is also consistent with the comparison of spectra 22 and 28 of Figure 3b revealing rather comparable intensities and similar line shapes, which were obtained from relatively darker and brighter sections of the same Pd disk shown in Figure 3c, respectively. It should also be noted that differences between the nano-FTIR signal intensities for the sulfates on the alumina domains and Pd domains (Figure 3a,b) may arise from the dissimilarities in the dipole moment strengths/alignments of different sulfate vibrational modes with respect to the tip–substrate.^61^

### Identification of the Chemical Nature of Adsorbates on Different Adsorption Sites

**Figure 4:**
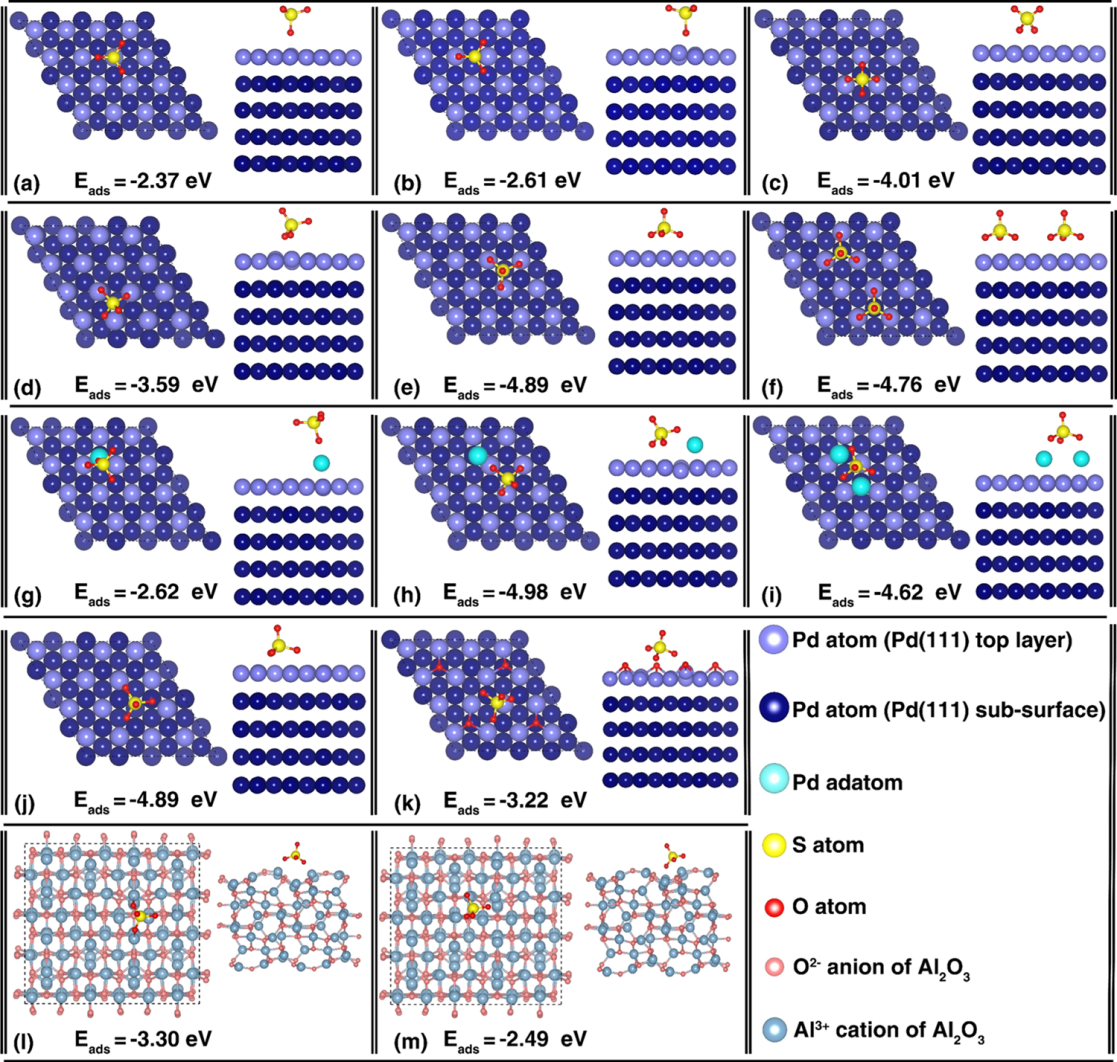
DFT calculation results and the corresponding average adsorption energies of some of the stable (top view and side view) adsorption configurations of sulfate species: (a) monodentate sulfate adsorbed on top of a Pd atom of Pd(111), (b) monodentate sulfate adsorbed between two Pd atoms of Pd(111), (c) bidentate sulfate adsorbed on two Pd atoms of Pd(111), (d) tilted sulfate adsorption on Pd(111), (e) tridentate sulfate adsorption on three Pd atoms of Pd(111), (f) tridentate sulfate adsorbed on Pd(111) next to a neighboring tridentate sulfate, (g) monodentate sulfate adsorption on a Pd adatom on Pd(111), (h) tridentate sulfate simultaneously adsorbed onto a Pd adatom and two Pd(111) surface atoms, (i) bidentate sulfate adsorbed on two Pd adatoms on Pd(111), (j) tridentate sulfate adsorption on a Pd atom monovacancy of Pd(111), (k) tridentate/bidentate sulfate adsorption on Pd atoms of the oxygen adatom-decorated Pd(111), (l) tridentate/bidentate sulfate adsorption on γ-Al_2_O_3_, and (m) alternative tridentate/bidentate sulfate adsorption on γ-Al_2_O_3_.

To shed light on the origins of the nano-FTIR spectroscopic features given in Figure 3, we performed ab initio density functional theory (DFT) calculations for various stable sulfate species on Pd and alumina surfaces (Figure 4).

DFT calculations for sulfate adsorption on clean Pd(111) single-crystal surfaces suggests that sulfate adsorbs extremely strongly on Pd(111), where the adsorption strength increases with increasing coordination of the sulfate group to the Pd(111) surface: *E*_ads_ (monodentate sulfate) < *E*_ads_ (bidentate sulfate) < *E*_ads_ (tridentate sulfate) (Figure 4a–e). Furthermore, Figure 4f shows that the adsorbate–adsorbate interaction is slightly repulsive and does not significantly alter the adsorption energy, indicating the possibility of high sulfate coverage under severe poisoning conditions (compare Figure 4f with Figure 4a–e). The presented configuration in Figure 4f is computed as the optimum distance between two adsorbates (5.05–5.31 Å between S atoms, and 2.99–3.14 Å between the closest O atoms based on several calculations carried out with varying initial geometries). This slightly repulsive interaction between the adsorbed sulfate atoms was also confirmed when the analysis was repeated with three sulfates, where *E*_ads_ was observed to decrease marginally to −4.64 eV.

Comparison of the monodentate sulfate adsorption strength on clean Pd(111) (Figure 4a, *E*_ads_ = −2.37 eV) with that of monodentate sulfate adsorption on a single Pd adatom on Pd(111) given in Figure 4g (*E*_ads_ = −2.62 eV, a simple model crudely mimicking a reconstructed and roughened Pd nanodisk surface) suggests that the generation of coordinatively unsaturated defect sites on Pd(111) can increase the adsorption strength of sulfates. This observation is also valid to a certain extent for higher sulfate coordination (e.g., compare tridentate sulfates on reconstructed Pd(111)-containing Pd adatom(s) given in Figure 4h,i with that of Figure 4d–f corresponding to the unreconstructed Pd(111) surface).

It is also apparent that the presence of other types of crystal defects such as Pd atom monovacancies (compare Figure 4j with Figure 4e,f) does not significantly alter the sulfate adsorption strength. In contrast, the existence of oxygen adatoms on Pd(111) weakens the adsorption energy of sulfates (compare Figure 4k with Figure 4e,f). It is worth mentioning that the currently computed adsorption energies for sulfates on Pd surfaces were also consistent with former DFT studies on sulfate adsorption on Pt(111).^44,62^ DFT calculations were also carried out on the γ-Al_2_O_3_ surface (Figure 4l,m), which revealed that sulfates also adsorbed strongly on alumina with tridentate/bidentate configurations.

The current DFT computational findings presented above imply that not only the adsorption configurations but also the adsorption strengths of sulfates on the Pd nanodisk/Al_2_O_3_ (thin-film)/Si(100) surface can be varied by altering the extent of sulfate poisoning by increasing the H_2_SO_4_(aq) exposure duration, which may in turn increase the sulfate surface coverage and modify Pd oxidation states and the nanodisk surface morphology via adsorbate-induced reconstruction and surface roughening.^63^ In other words, there may be a variety of dissimilar adsorbed sulfate species on Pd surfaces due to surface defects and imperfections. These implications will be demonstrated experimentally via additional nano-FTIR measurements presented in the forthcoming sections, revealing a variety of different sulfate vibrational signatures as a function of differences in adsorbate adsorption geometry, coordination, and adsorption site.

Overall, based on the current DFT calculations, it can be argued that sulfates can strongly adsorb on: (i) metallic (Pd^0^) adsorption sites of Pd(111) facets with monodentate/bidentate/tridentate configurations, (ii) defect sites of Pd nanodisks (e.g., Pd adatoms or Pd vacancies) in monodentate/bidentate/tridentate configurations, (iii) partially oxidized Pd^*x*+^ sites of oxygen-covered Pd(111) surfaces of Pd nanodisks with tridentate coordination, and (iv) the alumina surface with bidentate/tridentate configurations.

Thus, in light of the current DFT computational studies as well as the former experimental IRAS data obtained from low-index Pd single-crystal surfaces upon electrochemical H_2_SO_4_(aq) adsorption^34^ (see Figure S3, Table S1, and SI Section 4 for a detailed discussion) and former FTIR studies on the sulfur poisoning of high-surface-area powder catalysts,^4,38,39,52,64^ we can assign the currently observed nano-FTIR vibrational features presented in Figure 3.

Blue spectra given in Figure 3a,b were obtained from alumina domains of the mildly sulfur-poisoned Pd nanodisk/Al_2_O_3_ (thin-film)/Si(100) model catalyst surface. While the features of the blue spectra in Figure 3a,b observed at 1108, 1190, and 1306 cm^–1^ can be associated to bulk-like sulfates (i.e., Al_2_(SO_4_)_3_)^4,64^ (Figure 3e), vibrational features located at 1064,^39^ 1150,^39^ 1358,^4,64^ and 1432^4,64^ cm^–1^ on Al_2_O_3_ domains can be ascribed to surface sulfates with 2-fold (bidentate) adsorption geometry^40,51^ (Figure 3f). These assignments are also in line with our control experiments performed on a mildly sulfur-poisoned Al_2_O_3_ (thin-film)/Si(100) surface (Figure 3i, spectrum v) in the absence of any Pd adsorption site.

The major vibrational features of the mildly sulfur-poisoned Pd nanodisks appear within 1050–1450 cm^–1^ (purple spectra in Figure 3a,b). Accordingly, we identify the high-frequency shoulder at 1190 cm^–1^ as sulfates adsorbed with 3-fold or 1-fold geometry with the *C*_3*v*_ symmetry on electron-deficient Pd^*x*+^ sites (Figure 3g). Furthermore, we attribute the strongest feature at 1108 cm^–1^ to sulfates adsorbed with 3-fold geometry on metallic sites (Pd^0^) of Pd(111) facets (Figure 3h), while we assign the weaker shoulder at 1081 cm^–1^ to 1-fold sulfate adsorption on Pd(100)/Pd(110) facets with metallic Pd sites. Minor features at 1306, 1358, and 1432 cm^–1^ can be tentatively assigned to 2-fold or 3-fold sulfates adsorbed on oxidized Pd sites (i.e., Pd^*x*+^). Finally, we note that in the aforementioned electrochemical study,^34^ the utilized Pd surface was presumably more oxidized than the current work, leading to a slight blue shift in the observed vibrational features as compared to the current results.

To further corroborate these conclusions, we carried out a control experiment where we compared the near-field nano-FTIR spectra of a sulfur-poisoned Pd(nanodisk)/Al_2_O_3_ model catalyst with a conventional far-field attenuated total reflectance infrared (ATR-IR) experiment (Figure 3j) executed after an identical mild poisoning treatment. Despite the fact that far-field ATR-IR and near-field nano-FTIR techniques involve dissimilar spectroscopic absorption/reflection phenomena and different spectroscopic selection rules,^65,66^ the corresponding far-field ATR-IR spectroscopic data can be exploited to ensure the lack of entirely different vibrational signatures in the near-field nano-FTIR data. The far-field ATR-IR spectrum (Figure 3j) was acquired using a 2 mm × 2 mm ZnSe ATR crystal, which resulted in averaging of ca. >10^6^ Pd nanodisks on Al_2_O_3_ and thus revealed a clear convolution of sulfate signals originating from both the Pd and alumina domains. In stark contrast, the near-field nano-FTIR technique can resolve the spectral features characteristic to a single Pd nanodisk or Al_2_O_3_ sites on the model catalyst surface with nanometer-scale spatial resolution. We also note that the nano-FTIR data obtained from pristine (i.e., not sulfur-poisoned) Pd nanodisk or Al_2_O_3_ domains demonstrate that the nano-FTIR vibrational features obtained after mild sulfur poisoning (Figure 3i) are not due to background/spectroscopic artifacts. Finally, we mention that the corresponding ATR-IR spectrum of a pristine Pd/Al_2_O_3_ model catalyst lacks sulfate features and only shows IR absorption of the phonon features of the SiO*_x_* overlayer of the Si(100) substrate (SI Section 5 and Figure S4a). Thus, the dip observed in the ATR-IR spectrum (Figure 3i) of the sulfur-poisoned sample at 1108 cm^–1^ originates from inefficient background subtraction of this SiO*_x_* feature.

### Extreme Sulfur Poisoning, Reconstruction, and Roughening of Pd Nanodisks

**Figure 5:**
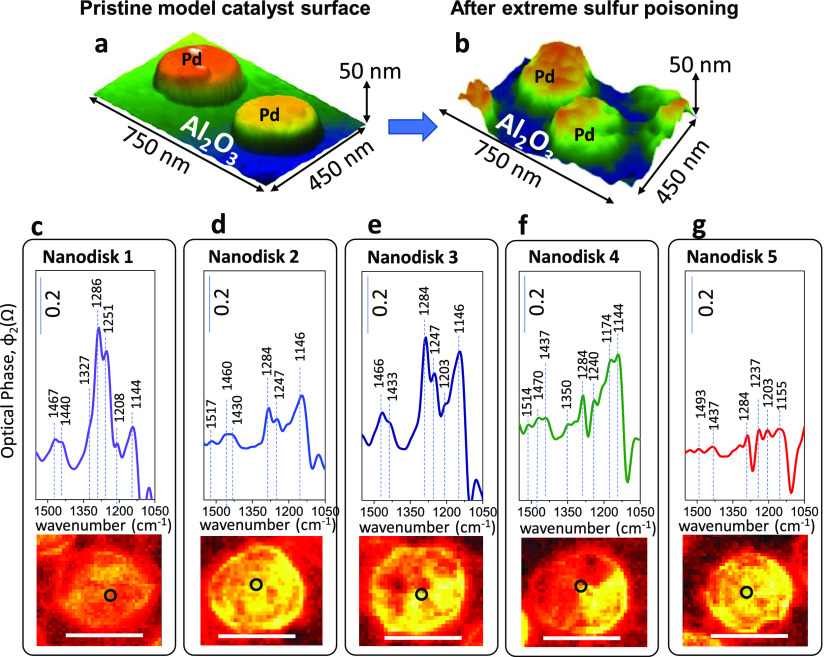
Demonstration of nanoparticle-to-nanoparticle variations in sulfate poisoning over Pd nanodisks on alumina thin films after extreme sulfur poisoning. (a, b) (False color) 3D AFM images of (a) pristine and (b) extremely sulfur-poisoned Pd(nanodisk)/Al_2_O_3_ (thin-film)/Si(100) model catalyst. (c–g) Nano-FTIR spectra obtained from five different extremely poisoned Pd nanodisks (spectra were collected at the points indicated by the black circles in the central zone of each nanodisk). The scale bar in the total IR-reflection s-SNOM images given at the bottom of each panel corresponds to 250 nm.

The current DFT studies and former experimental studies in the literature^63,67,68^ indicate that increasing the sulfate surface coverage may result in the enhancement of the nano-FTIR signals along with the modification of the surface morphology of the Pd nanodisks and alteration of the sulfate adsorption configurations. In addition, extensive exposure of the model catalyst to H_2_SO_4_(aq) can also lead to further oxidation of Pd sites. To demonstrate some of these aspects, in Figure 5, we investigate how the extent of sulfate poisoning and the adsorbed sulfate types vary from one Pd nanoparticle to another. As discussed earlier, the current XPS measurements (Figures 2e and S2a,b) indicate that sulfate poisoning of the model catalyst surface leads to the oxidation of Pd nanodisks. Increasing the extent of poisoning further accentuates this effect resulting in the observation of higher-frequency sulfate nano-FTIR features. Accordingly, nano-FTIR features appearing at 1240–1280 cm^–1^ in Figure 5 (which are absent on the mildly poisoned Pd nanodisks given in Figure 3a,b) can be ascribed to monodentate and/or tridentate sulfate adsorption on oxidized Pd^*x*+^ sites of the heavily reconstructed and roughened Pd nanodisks, whereas the highest-frequency sulfate features located within 1420–1511 cm^–1^ in Figure 5 can be attributed to bidentate and tridentate sulfate adsorption on fully oxidized Pd^2+^ sites.

Nano-FTIR spectroscopic variations observed among different Pd nanodisks given in Figure 5 clearly illustrate the drastic heterogeneity of the different PGM sites located on the same model catalyst surface, which were exposed to the identical poisoning treatment. For instance, comparison of the overall nano-FTIR signal intensities in Figure 5c,e,f with that of Figure 5d,g suggests that the nanodisks in the latter set are poisoned to a lesser extent. This is an interesting observation and a direct demonstration revealing that on the same catalyst sample, while some catalytically active PGM nanoparticles were severely poisoned, some other nanoparticles were almost not poisoned at all, after having been exposed to identical poisoning conditions. These striking near-field nano-FTIR spectroscopic results highlight how the structure and poisoning behavior of heterogeneous catalysts can actually be much more inhomogeneous than that typically inferred by conventional far-field spectroscopic measurements.

Furthermore, nano-FTIR spectra given in Figure 5c–g also indicate that not only the overall extent of sulfur poisoning but also the adsorption sites and adsorption configurations of sulfate species present on severely poisoned Pd nanodisks can be markedly different from each other. Along these lines, comparison of Figure 5c,f reveals that while vibrational features located within 1240–1280 cm^–1^ due to monodentate and/or tridentate sulfate adsorption on oxidized Pd^*x*+^ sites are dominant in the former case (i.e., Nanodisk 1), in the latter case (i.e., Nanodisk 4), signals within 1100–1174 cm^–1^ are prominent, which can be ascribed to sulfates adsorbed with 3-fold or 1-fold geometry on relatively less electron-deficient Pd^*x*+^ sites and/or metallic Pd^0^ sites.

Nanoparticle-to-nanoparticle variations in sulfur poisoning of different Pd nanodisks belonging to the same model catalyst sample given in Figure 5c–g can be due to the differences in the surface chemistry (i.e., morphology and electronic structure) of these different nanodisks such as the variations in the extent of the oxidation of the Pd sites and dissimilarities in the adsorbate-induced surface defects such as kinks, corners, step edges, etc., which may alter the sulfate adsorption strength and adsorption configuration as suggested by the current DFT results (Figure 4e,k). For instance, relatively weaker sulfate nano-FTIR features in Figure 5d,g as compared to the rest of the Pd nanodisks in Figure 5 can be associated to the greater number of coordinatively unsaturated Pd atoms adsorbing sulfates in a monodentate fashion (e.g., Figure 4g) or a greater surface oxygen coverage (e.g., Figure 4k). The current findings clearly illustrate the potential of the nano-FTIR technique, which can identify variations in the catalytic adsorbates/poisons on PGM active sites from one nanoparticle to another by eliminating the limitations of the ensemble averaging dictated by conventional far-field spectroscopies. Currently presented efforts may pave the way toward future investigations on “single-particle catalysis with high chemical definition”, which may allow us to study heterogeneous catalysis and heterogeneous catalytic poisoning phenomena on individual nanoparticles with vast number of structural and functional differences without compromising on the detailed adsorbate structure and adsorption configuration information.

**Figure 6:**
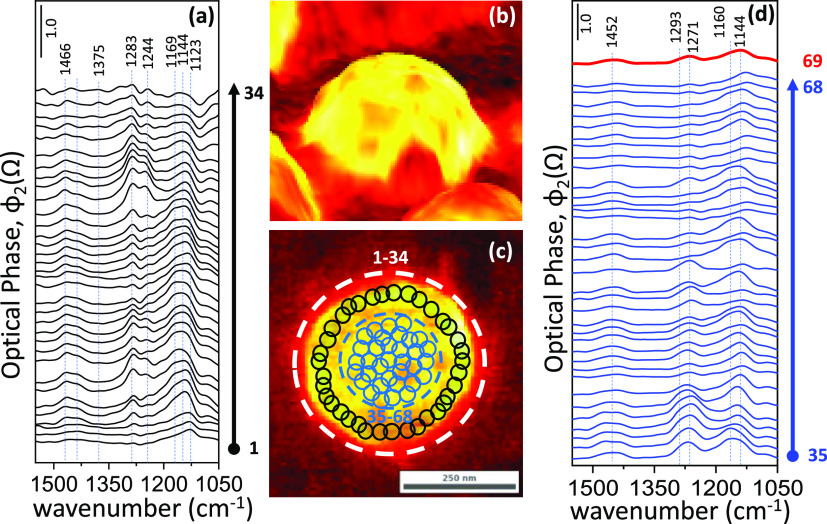
Demonstration of detailed variations of the sulfate species on a single Pd nanodisk after extreme sulfur poisoning of the Pd(nanodisk)/Al_2_O_3_ (thin-film)/Si(100) model catalyst surface. (a) Nano-FTIR data (spectra 1–34) obtained from locations close to the Pd/alumina interface (i.e., PGM catalytic active site/catalytic support heterojunction). (b) 3D and (c) 2D total IR-reflection s-SNOM images of the severely poisoned Pd nanodisk. (d) Nano-FTIR data (spectra 35–68) obtained from the center of the severely poisoned Pd nanodisk. Spectrum 69 on the top of panel (d) corresponds to the arithmetic average of spectra 35–68, mimicking the spectroscopic convolution that is typically observed in a far-field spectroscopic experiment.

As another illustration of the capabilities of nano-FTIR spectroscopy, we conducted a comprehensive nanometer-scale vibrational spectroscopic analysis of a single (roughened and reconstructed) Pd nanodisk after extreme sulfur poisoning (Figure 6). These experiments revealed two distinctly important findings. First, nano-FTIR spectra presented in Figure 6a,d clearly indicate that even on a single Pd nanodisk, there exist markedly different spectroscopic sulfate features revealing variations in adsorption types and adsorption sites of sulfate species. Second, one can directly investigate the PGM active site/catalytic support interface (Figure 6b,c) and observe the particular adsorbate types at this heterojunction, which might be critical in the catalytic reaction mechanisms of various processes.^69,70^ Comparative investigation of the nano-FTIR data given in Figure 6a (corresponding to PGM active site/catalytic support interface) with that of Figure 6d (associated with the central section of the roughened Pd nanodisk) suggests that at the PGM active site/catalytic support interface, two different sulfate species with nano-FTIR signatures at 1285 and 1247 cm^–1^ are observable (due to different monodentate and/or tridentate sulfate species on oxidized Pd^*x*+^ sites), while there exists only a single nano-FTIR signal at 1270 cm^–1^ on the roughened Pd nanodisk. This observation is in line with the increased heterogeneity of the adsorption sites at the PGM active site/catalytic support interface. Furthermore, comparison of the topmost (red) nano-FTIR spectrum given in Figure 6d (obtained by the arithmetic average of spectra 35–68, mimicking the spectroscopic convolution that is typically observed in a far-field spectroscopic experiment) and the far-field ATR-IR spectrum of an extremely poisoned Pd(nanodisk)/Al_2_O_3_ (thin-film)/Si(100) model catalyst surface (Figure S4b) indicates close resemblances, which are consistent with the fact that the far-field ATR-IR data is comprised of sulfate vibrational features originating from the convolution of the IR signals generated by numerous Pd disks.

Detailed AFM height profile (surface roughness) analysis of the pristine and mildly poisoned Pd nanodisks (Figure S5a,b) reveals rather comparable behavior, suggesting that mild sulfation leads to only minor reconstruction of the Pd(nanodisk)/Al_2_O_3_ (thin-film)/Si(100) model catalyst surface. This is also evident by the minor changes in the nano-FTIR spectra obtained from different parts of the same mildly poisoned Pd disk or from a different mildly poisoned Pd disk (Figure 3). In stark contrast, upon severe sulfation, AFM height profile analysis (Figure S5c) yields significant reconstruction of the model catalyst surface, illustrating extended corrugations on Pd disk terraces and increased curvature of the Pd nanodisks along with significant variations in the nano-FTIR spectra not only obtained from a single Pd disk (Figure 5) but also from different Pd disks (Figure 6). These findings indicate that the variations in the nano-FTIR spectral fingerprints upon extreme sulfation can be, in part, linked to the alterations in the sulfate adsorption configurations and adsorption sites due to surface roughening, defect formation, and reconstruction.

As a second factor, variations in the oxidation state of the surface Pd sites can also contribute to the observed inhomogeneities in the current nano-FTIR spectra (Figures 5 and 6), resulting in the formation of a variety of sulfate adsorption configurations. XPS data presented in Figure S2a,b show that while Pd^0^ sites are by far the most predominant species (Pd^0^/Pd^2+^ = 3.2) on clean Pd nanodisks, relative abundance of Pd^2+^ species increased monotonically with increasing extent of sulfation so that on the severely poisoned model catalyst surface, Pd^2+^ species became the most prominent species (Pd^0^/Pd^2+^ = 0.8).

It is important to note that diversity in the vibrational features presented in Figures 5 and 6 does not originate from spectroscopic artifacts due to the sample height-dependent (i.e., roughness-related) variations in the near-field strength but are due to the chemical contrast. Unlike the sample height-dependent alterations reported in a recent study carried out on films of poly(4-vinylpyridine) (P4VP) with ca. 7° angle of elevation on gold and silicon substrates,^71^ our control experiments (Figure S6) clearly showed that there is no direct correlation between sample height/roughness and the observed nano-FTIR spectral line shapes or intensities. Thus, the currently presented nano-FTIR spectra are due to chemical contrast rather than spectroscopic artifacts. For instance, Figure S6 demonstrates that on the Pd nanodisks, two separate points with almost identical heights (i.e., heights differing by ≤0.36 nm) lead to notably different spectral line shapes and different intensity ratios between various vibrational features. On the other hand, Figure S6 also illustrates that two separate points on the same Pd nanodisk yield almost identical nano-FTIR spectra even though their heights differ by up to 15.4 nm. These findings are in very good accordance with the former study of Wang et al.,^71^ suggesting that near-field signal fluctuations leading to uncertainty in the chemical contrast are only significant when the roughness is comparable to the sample thickness.

### Catalyst Regeneration

In many industrial processes, negative effects of catalyst deactivation can be eliminated via particularly designed regeneration protocols that are carried out in a periodic fashion during catalyst operation. This is also the case for catalytic deactivation by sulfur, where the adsorbed poisoning SO*_x_* species can be converted into weakly bound volatile molecules, such as SO_2_, SO_3_, and H_2_S, with the help of various reducing agents, such as H_2_.^55^ Accordingly, desorption of these weakly bound reduced species frees up the active metal sites, as well as the poisoned domains on the support, rendering them available for the next catalytic cycle.

**Figure 7:**
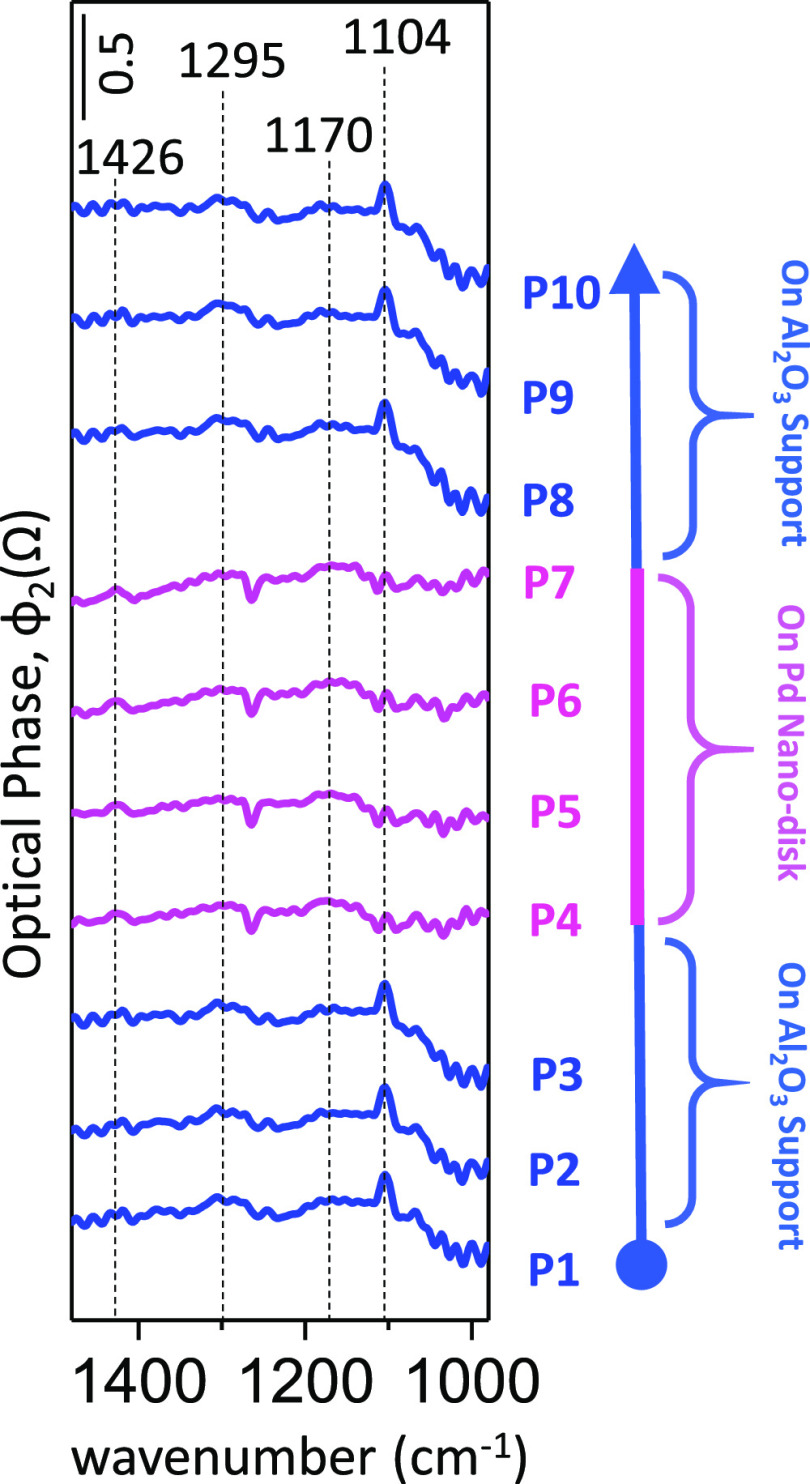
Nano-FTIR spectra obtained along a regenerated mildly poisoned Pd nanodisk on a Pd(nanodisk)/Al_2_O_3_ planar model catalyst surface after catalytic regeneration with H_2_(g) (4% H_2_(g) flow, 50 mL/min), 573 K, 2 h. Spectra labeled as P1–P3 and P8–P10 (blue) were directly acquired from Al_2_O_3_ domains, while the spectra in P4–P7 (purple) were directly acquired from the Pd nanodisk.

Thus, as a final demonstration of our experimental approach, we investigate the effect of the regeneration of a mildly poisoned Pd(nanodisk)/Al_2_O_3_ model catalyst given in Figure 3 with H_2_ at 573 K. The corresponding nano-FTIR spectra (Figure 7) reveal that most of the poisoning species can be removed from the catalyst surface by the regeneration treatment, leaving only residual sulfate species with extremely weak nano-FTIR signatures. Corresponding XPS data (Figure S2c,d) indicate that sulfur concentration on the surface increases with the extent of poisoning, followed by a significant decrease after the regeneration of both mildly and severely poisoned model catalysts. Furthermore, Pd 3d XPS results (Figure S2a,b) show that upon regeneration of the mildly poisoned Pd nanodisks, relative abundance of Pd^0^/Pd^2+^ surface sites become comparable to that of the clean Pd nanodisks, in line with a rather reversible surface oxidation/reduction of the Pd sites due to mild sulfation/regeneration. In contrast, extreme poisoning of the Pd nanodisks leads to relatively irreversible bulk oxidation of the Pd nanodisks, where Pd sites cannot be efficiently reduced even after regeneration (at least under the currently utilized reduction conditions), despite the removal of most of the surface sulfate species. This latter observation is also consistent with the corresponding far-field ATR-IR spectra obtained after the regeneration of mildly/extremely poisoned model catalysts (Figure S4b). Furthermore, AFM height profile analysis (Figure S5) of clean, mildly, and severely poisoned Pd nanodisks as well as their regenerated forms after poisoning indicated that the currently used regeneration protocol had a minor effect on the Pd nanodisk surface roughness. Significant differences in the relative nano-FTIR spectral line shapes and intensities before (Figure 3) and after (Figure 7) the regeneration of the mildly poisoned Pd nanodisks despite rather invariant surface roughness and comparable Pd nanodisk topography indicate that the currently observed nano-FTIR features are of chemical origin rather than spectroscopic artifacts associated with the sample height-dependent near-field signal alterations.

Figure 7 shows that after regeneration of the mildly poisoned model catalyst, alumina domains display vibrational features at 1104, 1174, and 1295 cm^–1^, which can be assigned to bulk-like sulfates (i.e., Al_2_(SO_4_)_3_), while Pd nanodisks exhibit miniscule features at 1170 and 1426 cm^–1^, which can be attributed to 3-fold/1-fold sulfates on Pd^*x*+^ and 2-fold/3-fold sulfates on Pd^*x*+^ sites, respectively. It is apparent that while all of the nano-FTIR signals obtained after regeneration are small, vibrational features on Pd nanodisks are even smaller than those of the ones on alumina domains. This may be tentatively attributed to the fact that during the initiation of the regeneration process with H_2_, hydrogen activation and formation of H-atoms occur on the Pd active sites, followed by spillover of H-atoms to the Al_2_O_3_ support domains. Therefore, it is likely that most of the Pd sites were almost fully regenerated during the reduction with H_2_, while some of the resilient bulk-like Al_2_(SO_4_)_3_ species survived.

Our findings point to an important general aspect of sulfur poisoning and regeneration of metal/metal-oxide catalytic interfaces. It can be argued that during the regeneration treatments of sulfur-poisoned metal/metal-oxide catalytic interfaces, while the PGM sites can be regenerated more effectively (as they possess the necessary sites for activating reducing agents), metal-oxide support sides that cannot directly activate reducing agents are regenerated relatively poorly. This may lead to the buildup of sulfur species on the metal-oxide support sites followed by migration of these high-surface-coverage poisoning species to the PGM sites, eventually rendering the latter incapable of being regenerated. Thus, future sulfur-tolerant catalytic metal/metal-oxide nanomaterial designs may focus on strategies enabling not only enhanced activation of reducing agents but also on approaches boosting the surface transport and diffusion of cleaved reducing species from the PGM active sites to the metal-oxide support sites in their extended proximity.

## Conclusions

In summary, our work demonstrates that the investigation of shape-defined uniform two-dimensional model catalysts with s-SNOM-based nano-FTIR spectroscopy enables important opportunities to study catalytic phenomena at the nanometer scale without sacrificing chemical information. As we have shown, this effective and versatile experimental approach allows the direct investigation of catalytic nanosystems at mid-IR wavelengths (i.e., 4.5–15.4 μm) and is able to reveal invaluable local chemical information, which to date, was not accessible using conventional spectroscopic characterization techniques. The current findings indicate that the types of adsorbates and their adsorption configurations on the catalyst surface may show significant variations not only on a single PGM nanoparticle but also among different PGM nanoparticles due to dissimilarities in the surface morphology (e.g., roughness, surface defects, and surface reconstruction) and differences in the local electronic structure (i.e., oxidation state) of the PGM adsorption sites.

## Methods

### Lithographic Model Catalyst Preparation

Details of the 2D model Pd(nanodisk)/Al_2_O_3_ (thin-film) catalyst preparation via the hole-mask lithography technique are summarized in SI Section 2. Additional details can be found elsewhere.^30^

### Sulfur Poisoning and Catalytic Regeneration Protocols

Sulfur poisoning was performed by immersing the catalyst into a diluted (1 × 10^–2^ M) H_2_SO_4_(aq) solution (Sigma Aldrich, 99.999% purity) for 10 min at 295 K to adsorb sulfuric acid anions on the model catalyst surface. Then, the sample was dried on a hot plate in air at 383 K for 10 min. Catalytic H_2_-desulfation/regeneration experiments were performed on the sulfur-poisoned model catalysts via annealing in a tube furnace at 573 K in 4% H_2_(g) flow with a 50 mL/min flow rate for 2 h. Extreme poisoning experiments leading to morphological alterations of the model catalyst surface were carried out by immersing the catalyst into a diluted (1 × 10^–2^ M) H_2_SO_4_(aq) solution for 24 h at 295 K. Then, the sample was dried on a hot plate in air at 383 K for 10 min. The reduction temperature used in the regeneration protocol was chosen as the highest temperature allowing maximum regeneration while preserving the morphology of the 2D model Pd(nanodisk)/Al_2_O_3_ (thin-film) catalyst.

### Nano-FTIR Spectroscopy and s-SNOM Imaging

s-SNOM/nano-FTIR analyses were performed using a neaSNOM instrument manufactured by Neaspec GmbH, Germany, with a spectral range of 650–2200 cm^–1^ (i.e., 15.4–4.5 μm). The interferogram signal was collected via the backscattered IR radiation from the SNOM tip surface and recorded by the demodulated detector. In the current manuscript, phase spectra were utilized to obtain the nano-FTIR spectra. Nano-FTIR spectra were acquired by averaging 20 interferograms with 2.5 cm^–1^ spectral resolution for 3.5 min total acquisition time. All spectra were collected within a frequency range of 840–1650 cm^–1^. A clean Pd(nanodisk)/Al_2_O_3_ (thin-film) model catalyst surface was used to obtain the background nano-FTIR spectrum. *Y*-axes of the nano-FTIR spectra given in this work show the second harmonic of the imaginary component (phase, φ_2_(Ω)) of the scattered s-SNOM signal, where the SNOM tip was operated in tapping mode with a mechanical cantilever resonance frequency of Ω = 250–270 kHz and a tapping amplitude of about 80 nm, while the scattered s-SNOM signal demodulation was carried out at the second harmonic (2Ω) to suppress the non-near-field (or far-field background) spectroscopic artifacts. Former studies in the literature showed that the phase of the second harmonic of the scattered s-SNOM near-field signal corresponded to the nano-FTIR absorption spectrum of the sample.^19,22,44^ The reader can refer to SI Section 1 for more information.

### SEM and EDX Analyses

A Carl Zeiss EVO40 environmental SEM equipped with a LaB_6_ electron source and a Bruker AXS XFlash 4010 detector was used for SEM and EDX analyses with a 15 kV electron acceleration voltage, where the analyzed samples were attached to electrically conductive carbon films placed on aluminum stubs.

### ATR-IR Measurements

ATR-IR spectra were acquired using a Bruker Alpha FTIR spectrometer equipped with an ATR module containing a ZnSe ATR crystal and a deuterated triglycine sulfate (DTGS) mid-IR detector. ATR-IR spectra were recorded by averaging 128 scans with a 4 cm^–1^ spectral resolution. A clean Pd(nanodisk)/Al_2_O_3_ (thin-film) model catalyst surface was used in the recording of the background ATR-IR spectra.

### XPS Measurements

XPS analyses were performed via a SPECS PHOIBOS hemispherical energy analyzer. A monochromatic Al Kα X-ray excitation source (14 kV, 350 W) and an electron flood gun were employed during the XPS data acquisition for charge neutralization. Binding energies were calibrated using the C 1s surface carbon signal at 284.8 eV. XPS spectra were fitted using Casa XPS software via Shirley background subtraction and mixed Gaussian–Lorentzian peak shapes for the deconvolution of Pd 3d signals.

### Density Functional Theory Modeling Studies

All of the ab initio calculations were carried out within the framework of density functional theory (DFT)^72,73^ using the Vienna Ab initio Simulation Package (VASP).^74−77^ The projector-augmented wave (PAW)^78^ method was employed to describe the element potentials. The Perdew–Burke–Ernzerhof (PBE)^79^ functional form of generalized gradient approximation (GGA) was adopted to describe the exchange and correlation potential. The kinetic cutoff energy for plane waves was taken as 520 eV. To represent the Pd(111) and γ-Al_2_O_3_(110) surfaces, 4 × 4 × 1 and 2 × 2 × 1 supercells with five- and eight-layered slabs were constructed, respectively. These thicknesses are tested to obtain converged surface energies. The lattice parameters of slabs are optimized and calculated as 10.92 Å × 10.92 Å and 17.30 Å × 15.84 Å for Pd(111), and γ-Al_2_O_3_(110), respectively, which are also in agreement with the literature.^80,81^ For structural relaxation and adsorption calculations of sulfate species, the convergence criteria for the total energy and allowed force on atoms were considered to be 10^–5^ eV and 0.01 eV/Å, respectively. The Brillouin zone (BZ) was sampled using Γ-centered 4 × 4 × 1 *k*-point meshes for both the slabs based on the Monkhorst–Pack scheme.^82^ The van der Waals interactions were taken into account by implementing the DFT-D3 method. The dipole correction was taken into account along the surface normal direction.^83^ A vacuum spacing of 12 Å was inserted to prevent spurious interactions due periodic boundary conditions. To estimate the interaction strength between the molecule and the surfaces, the adsorption energy per adsorbate is defined as
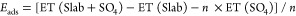
where ET (Slab + SO_4_), ET (Slab), and ET (SO_4_) are the total energy of the slab with adsorbed SO_4_, Pd(111), or Al_2_O_3_ slab (with or without defects), and SO_4_ species, respectively. *n* indicates the number of adsorbed SO_4_ species. Atoms in the bottom three layers of the slabs were fixed during the adsorption studies.

## Supporting Information Available

Nano-FTIR spectroscopy instrumental details and measurement parameters (Section S1), detailed description of sample preparation and hole-mask lithography (Section S2), XPS analysis details and additional XPS data (Section S3), literature survey and summary of current results on spectroscopic assignments of the nature of S-related adsorbates (Section S4), far-field ATR-IR measurements (Section S5), and AFM height profile measurements and control experiments demonstrating the lack of a direct correlation between surface roughness and nano-FTIR features (Section S6) (PDF)
