# Supplementary material for: Unraveling Molecular Fingerprints of Catalytic Sulfur Poisoning at the Nanometer Scale with Near-Field Infrared Spectroscopy
Source: J Am Chem Soc. 2022 Apr 29;144(19):8848–60. doi: 10.1021/jacs.2c03088 (PMC9121382; doi:10.1021/jacs.2c03088)
Supplement: Supplementary file 1 — ja2c03088_si_001.pdf [file ja2c03088_si_001.pdf]

Unraveling Molecular Fingerprints of Catalytic Sulfur Poisoning at the Nanometer Scale with Near-Field Infrared Spectroscopy

Zafer Say[†, ¥, ‡], Melike Kaya[†, Ұ], Çağıl Kaderoğlu[Ұ, ₮], Yusuf Koçak[†], Kerem Emre Ercan[†], Abel Tetteh Sika-Nartey[†], Ahsan Jalal[†], Ahmet Arda Turk[†], Christoph Langhammer[‡], Mirali Jahangirzadeh Varjovi [₳], Engin Durgun[₳], and Emrah Ozensoy[†,₳, *]

[†]*Department of Chemistry, Bilkent University, 06800, Ankara, Turkey*

[¥]*Department of Materials Science and Nanotechnology Engineering, TOBB University of Economics and Technology, 06510, Ankara, Turkey*

[‡]*Department of Physics, Chalmers University of Technology, SE-412-96 Gothenburg, Sweden*

[†] *Institute of Acceleration Technologies, Ankara University, Ankara 06830, Turkey*

[Ұ]*Turkish Accelerator and Radiation Laboratory (TARLA), 06830 Ankara, Turkey.*

[₮]*Department of Physics Engineering, Ankara University, 06100 Ankara, Turkey.*

[₳]*UNAM - National Nanotechnology Research Center, Bilkent University, 06800, Bilkent, Ankara, Turkey*

Supporting Information:

1. Scattering Type-Scanning Near Field Optical Microscopy (s-SNOM) and Near-field Nano infrared Spectroscopy (nano-FTIR) Measurements

s-SNOM and nano-FTIR measurements were performed using a NeaSpec neaSNOM microscopy/spectroscopy set-up by illuminating the s-SNOM tip with a coherent broadband mid-IR (MIR) source generating an average output power of ca. 1 mW, produced by a difference frequency generator (DFG). This MIR source provided the analysis of a spectral window within the frequency range of 650-2200 cm^{-1}. Nano-FTIR interferogram signal was collected using the back-scattered IR radiation from the s-SNOM tip surface and recorded by the detector followed by the signal demodulation at the second harmonic of the natural oscillation frequency of the s-SNOM tip. In the current work, nano-FTIR spectra was based on the phase spectra of the back-scattered IR radiation. All nano-FTIR spectra were collected to cover the frequency range within 840-1650 cm^{-1} which is the relevant MIR spectral region for sulfates. Nano-FTIR background spectra were acquired from the clean Pd(nano-disk)/Al_2O_3 model catalyst surface. These background spectra were subtracted from the sample nano-FTIR spectra to eliminate background spectral artifacts originating from the clean sample surface and to emphasize the vibrational spectroscopic features of the adsorbate overlayers.

2. Hole-mask Colloidal Lithographic Model Catalyst Synthesis:

An oxidized Si(100) wafer was used as the substrate. Before the lithographic manufacturing of the two dimensional Pd(nano-disk)/Al_2O_3 (thin film)/Si(100) model catalyst,[1] oxidized Si wafer substrate was thoroughly cleaned with acetone and isopropanol (IPA) in an ultrasonic bath at 40 °C. Then, Al_2O_3 thin film was grown on the substrate via e-beam physical vapor deposition using a Kurt Lesker PVD 225 deposition system to obtain an alumina thin film with a thickness of 300 nm (Figure S1). X-ray diffraction (XRD) analysis of the alumina film (data not shown) revealed no diffraction signals suggesting the presence of a disordered alumina thin film. Deposition rate used in the Al_2O_3 PVD process was 1 Å/s, where the deposition rate and the associated final film thicknesses were controlled using a quartz crystal microbalance (QCM) at a chamber base pressure of < 10^{-7} Torr. Note that an identical deposition rate was used for the coating of Pd and Au layers that are described below. Next, poly(methyl methacrylate) (PMMA) sacrificial layer was spin coated onto the Al_2O_3 thin film followed by drying on a hot plate resulting in a polymeric film with a thickness of ca. 200 nm. Then, positively charged poly (diallyldimethylammonium chloride) (PDDA, Sigma Aldrich, diluted in 20% H_2O) aqueous solution was dispersed onto the PMMA layer after surface treatment in an oxygen plasma chamber. This was followed by the removal of the excess PDDA solution via blow drying. Next, negatively charged polystyrene (PS, Life Technology, diluted in water) nanobeads with an average diameter of 250 ± 20 nm were introduced on the sample surface and incubated for 2 min. Excess PS beads were subsequently washed and the surface was subsequently dried under N_2(g) flow. After that, a 15 nm-thick Au masking layer was grown over the sample surface via e-beam PVD in the Kurt Lesker PVD 225 system. Obtained material assembly containing PS beads and Au mask was tape-stripped to remove the nanobeads which resulted in the generation of holes on the Au mask with well-defined sizes characterized by the diameter of the nascent PS beads. These holes were then extended to underlying layers (i.e., thorough PDDA and PMMA layers, all the way down to the Al_2O_3 layer) using oxygen plasma etching. Next, Pd metal was evaporated onto the sample surface *via*

e-beam PVD in order to obtain a 50 nm-thick Pd layer. As the final step, all of the surface layers on the Pd(nanodisk)/Al_2O_3 (thin film)/Si(100) system were removed in a lift-off process by dissolving the PMMA layer in acetone followed by surface cleaning with IPA.

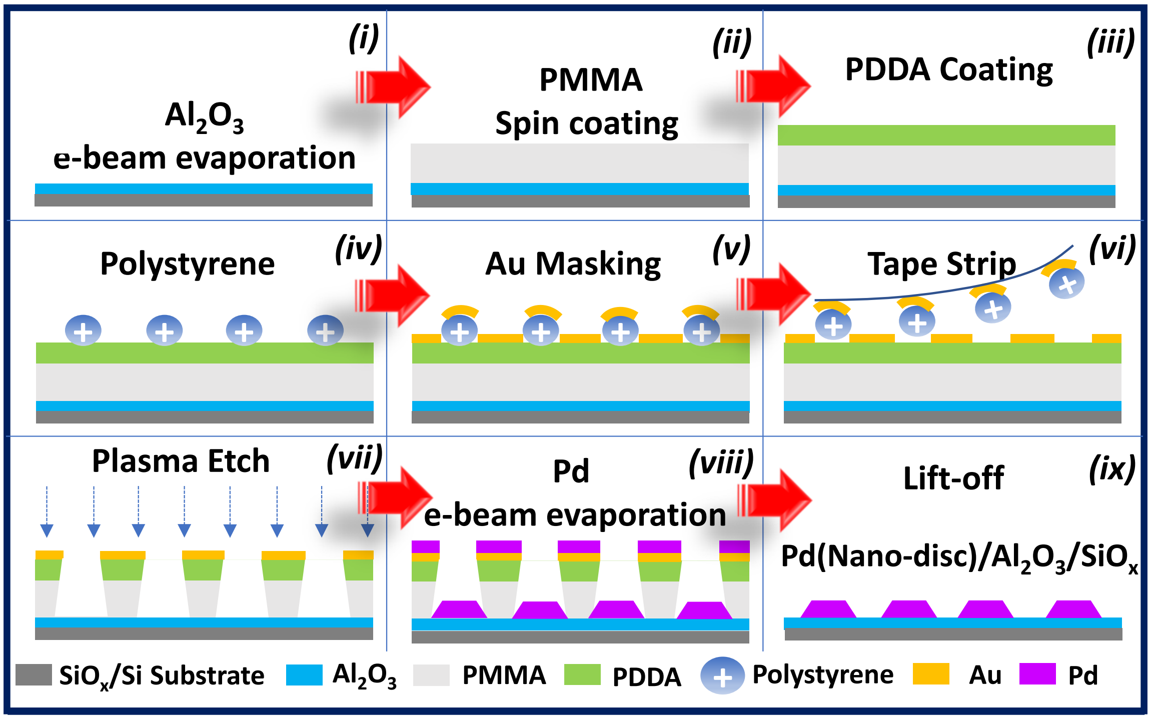

Figure S1. Schematic representation of lithographic manufacturing steps used in the nanofabrication of the 2D-Pd(nanodisk)/Al_2O_3 (thin film)/Si(100) model catalyst surface.

3. X-Ray Photoelectron Spectroscopy (XPS) Measurements:

XPS measurements were carried out using a SPECS PHOIBOS (SPECS GmbH, Germany) hemispherical energy analyzer. A monochromatic Al-K_α X-ray excitation source (14 kV, 350 W) and an electron flood gun (for charge neutralization) were employed in the XPS data acquisition, where the base pressure of the XPS chamber was < 1.0×10^{-9} Torr. Binding energy (B. E.) values in the XP spectra were calibrated using the C1s signal of the adventitious (surface) carbon species located at 284.8 eV. XP spectra were analyzed using Casa XPS software for Shirley background subtraction and peak deconvolution/fitting utilizing mixed Gaussian–Lorentzian peaks.

Detailed XPS analysis of the i) clean, ii) mildly sulfated, iii) mildly sulfated and subsequently regenerated, iv) extremely sulfated, and v) extremely sulfated and subsequently regenerated Pd(nanodisk)/Al_2O_3 (thin film) model catalyst surfaces are given in Figure S2. Pd3d XP spectrum of the pristine Pd(nanodisk)/Al_2O_3 (thin film) surface (Figure S2a) yielded two doublets revealing Pd3$d_{5/2}$ features at 335.8 eV and 337.3 eV, corresponding to metallic (Pd^0) and oxidized (Pd^{+2}) species, respectively.[2] Corresponding Pd3$d_{5/2}$ features of the sulfur poisoned Pd(nanodisk)/Al_2O_3 (thin film) model catalyst surface shifted to lower B. E. values of 335.2 eV (Pd^0) and 336.1 eV (Pd^{+2}) which can tentatively be attributed to the increasing number of point defects on the Pd nanodisk surfaces upon sulfur poisoning.[3,4] Furthermore, relative abundances of Pd^0 to Pd^{2+} species on the Pd(nanodisk)/Al_2O_3 (thin film) model catalyst surface before and after sulfur poisoning suggests that relative abundance of oxidic Pd species increases with the increasing extent of sulfur poisoning (Figure S2b). Figure S2b illustrates that while the relative amount of Pd^0 to Pd^{2+} species can be fully recovered after mild sulfation and subsequent regeneration; extreme sulfation leads to rather irreversible oxidation which is persistent even after regeneration. Surface atomic concentration % results obtained from XPS measurements (Figure S2c) indicate that surface S concentration increases with the extent of sulfation and decreases after the regeneration protocols without fully disappearing. Figure S2d illustrates the S2p region of the XP spectra. As expected, no sulfur related XPS features were detected on the clean sample surface, while sulfur-poisoned surface yielded a single S2p feature located at 170.2 eV revealing the presence of S^{6+} species (i.e., sulfates).[5-6] Al2p XP spectra (Figure S2e) indicated that Al2p XPS intensities are inversely proportional to the surface sulfate coverage (i.e., as S2p signal increases, Al2p signal decreases) which is consistent with the fact that sulfate species not only poison Pd species but also poison alumina sites. However due to the small spin-orbit splitting of the Al2p signals and the well-known convolution of the Al2$p_{1/2}$ and

Al2p$_{3/2}$ features, further analysis of the Al2p signals is not attempted. Finally, presence of sulfates also led to broadening and blue-shift of the O1s features (Figure S2f). However due to the complex overlap between O1s features originating from oxidized Pd, alumina and sulfate species, we did not carry out deconvolution of the O1s spectra.

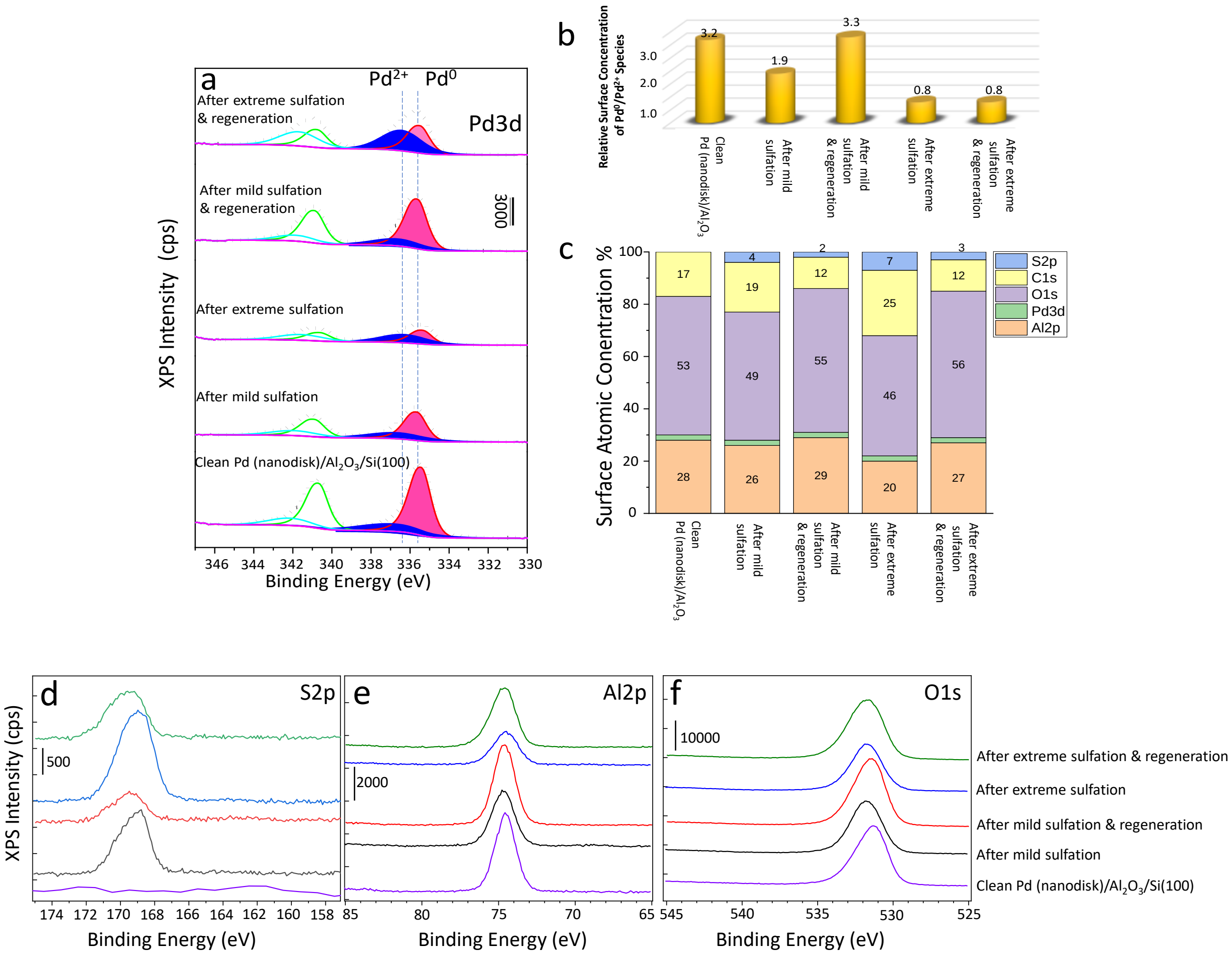

Figure S2. XPS analysis of the Pd(nanodisk)/Al_2O_3 model catalyst in its: clean form, after mild sulfation, after mild sulfation and subsequent regeneration, after extreme sulfation, and after extreme sulfation and subsequent regeneration. (a) Pd3d XP spectra, (b) relative extent of Pd^0/Pd^{2+} species obtained from (a), (c) Surface atomic concentration% values deduced from XPS analyses, (d) S2p, (e), Al2p, and (f) O1s XP spectra.

4. Spectroscopic assignment of the nature of the adsorbates generated upon H_2SO_4(aq) adsorption on Pd(nanodisk)/Al_2O_3 model catalyst surface:

As to whether SO_4^{2-} and/or HSO_4^- species exist on the Pd (nanodisk)/Al_2O_3 model catalyst surface after H2SO4(aq) adsorption and subsequent thermal treatment, it can be argued that SO_4^{2-} species are likely to be the predominant poisoning species. This is because, nano-FTIR spectroscopic features observed in the current work reveal significant similarities to that of the former in-situ IR spectroscopic studies, where SO_2(g) + O_2(g) was introduced on dehydroxylated forms of γ-Al_2O_3[6] or Pd/γ-Al_2O_3[7–9] yielding sulfates as the ultimate poisoning species. In these former in-situ IR spectroscopic studies, presence of HSO_4^- (ads) was unlikely due to i) the lack of H-containing species in the poisoning gas stream which contained only SO_2(g) + O_2(g), ii) absence of -OH functionalities (i.e. H-sources that can protonate sulfates) on the catalyst surface as a result of dehydroxylation attained in the high temperature pretreatment and poisoning steps; iii) thermal/catalytic decomposition of HSO_4^- at elevated temperatures to the more stable SO_4^{2-} species. Furthermore, lack of any HSO_4^- (ads) species on single crystal and polycrystal Pt surfaces upon H_2SO_4(aq) adsorption[10,11] is also in very good accordance with our current assignment. Since we establish the chemical composition of the generated poisoning species on the Pd (nanodisk)/Al_2O_3 model catalyst as mostly sulfates (i.e. SO_4^{2-}), we can provide further insight regarding their adsorption sites and adsorption configurations.

Sulfuric acid adsorption on Pt surfaces with different crystallographic orientations in electrochemical aqueous systems revealed typically either one or two vibrational bands located within 1000-1450 cm^{-1}. On the Pt(111) surface, only a single band

around 1200 cm^{-1} was reported, whose frequency varied with adsorbate coverage.[10,12–18] This feature was originally assigned to either HSO_4^- or SO_4^{2-} adsorbed on Pt(111)[12–18] with a 3-fold (tridentate) geometry revealing a C_{3v} symmetry, while a recent SFG study utilizing isotopically labeled D_2SO_4 (in D_2O) adsorption on Pt(111) clearly demonstrated that only sulfate species were present on the Pt(111) surface.[10] H_2SO_4(aq) adsorption studies on Pt(100) and Pt(110) surfaces yielded two vibrational features around 1100 and 1200 cm^{-1} which can be attributed to SO_4^- bound to the surface via 2-fold (bidentate) geometry with C_{2v} symmetry. 1100 cm^{-1} feature can be assigned to the vs (S-O*) band of SO_4^{2-} coordinated to Pt (with a longer bond length and reduced orbital overlap, leading to a red-shift in frequency) and the 1200 cm^{-1} band can be ascribed to the unco-ordinated vs (S-O) band of sulfates.[15] H_2SO_4(aq) adsorption on stepped Pt single crystal surfaces such as Pt(S)-[n(111) × (111)] led to anions on terraces with 3-fold geometry (1200 cm^{-1}) and on steps with 2-fold geometry (1200 and 1100 cm^{-1}), where the intensity of the 1100 cm^{-1} feature increased with increasing step density.[19] Same study showed that on stepped Pt(S)-[n(100) × (111)] single crystal surfaces, only 2-fold adsorption (1200 and 1100 cm^{-1}) was detected for both terraces and steps. On the other hand, very recently, detailed X-ray absorption spectroscopy (XAS) and computational modeling studies[11] regarding H_2SO_4(aq) adsorption on polycrystalline Pt electrodes (exhibiting various crystallographic orientations) revealed that only sulfate species were present on this Pt surface under electrochemical conditions and no evidence was found for the existence of HSO_4^- (ads) or Pt-O/Pt-OH (surf) species.

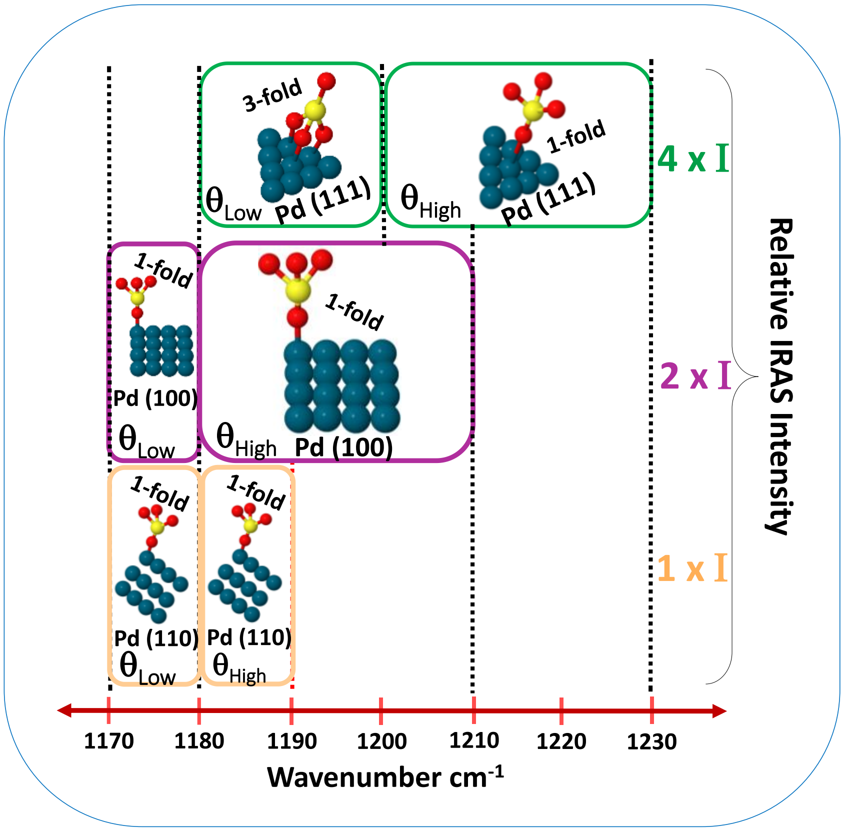

Figure S3. Summary of the characteristic vibrational features observed in former IRAS studies [generated using the data reported in Ref 12] investigating H_2SO_4(aq) adsorption on various Pd single crystal surfaces (θ_{low} and θ_{high} stand for low and high surface coverage, respectively).

H_2SO_4(aq) adsorption studies on Pd single crystal surfaces[12,20] in aqueous electrochemical systems suggested that on Pd(111), a single peak within 1180-1230 cm^{-1} was observed revealing C_{3v} symmetry. This peak was attributed to either HSO_4^- or SO_4^{2-} (and referred as the sulfuric acid anion without a precise identification) adsorbed via 3-fold geometry at low adsorbate coverages and 1-fold (monodentate) geometry at high coverages (both with C_{3v} symmetry) in an upright configuration (i.e. not tilted)[12]. In case of tilting of such adsorbates, due to the decreasing symmetry and loss in degeneracy, an additional band of the forbidden vibration vas(S-O) at a wavenumber higher than vs(S-O) is expected to appear.[20] On Pd(100), a single band with 1170-1210 cm^{-1} was detected at various coverages corresponding to 1-fold adsorption of sulfuric acid anion.[12] On stepped Pd single crystal surfaces with narrow terraces such as Pd(110) and Pd(311) only 1-fold adsorption (1180-1200 cm^{-1}) of the sulfuric acid anion was observed[12], while stepped surfaces with wider terraces such as Pd(S)-[n(111) × (111)] (n= 2, 3, 5, 9, 20) revealed both 1-fold and 3-fold adsorption (1175-1235 cm^{-1}) with C_{3v} symmetry.[20] These vibrational features were also reported to shift to higher frequencies with increasing adsorbate coverages and increasing oxidation state of the

PGM sites[12]. Note that the oxidation state of the Pd single crystals investigated in these former electrochemical studies were not reported and are likely to be different than the currently investigated Pd nanodisks in our work. Furthermore, for a given coverage, relative IRAS intensities of the sulfuric acid anions adsorbed on different Pd single crystal surfaces were ranked approximately in the following manner: IPd(111) ≈2×IPd(100) ≈ 4 IPd(110)/Pd(311).[12, 20] Vibrational features observed upon H_2SO_4(aq) adsorption on various Pd single crystal surfaces in former studies are summarized in Figure S3.

Liu et al. reported that H_2SO_4 adsorption on mesoporous γ-Al_2O_3 powders yielded convoluted and broad IR features which were attributed to OH-bending mode of hydrated alumina (1013 cm^{-1}), surface sulfates (1068 and 1176 cm^{-1}), and 3-fold sulfates (1283 cm^{-1})[21]. Former studies regarding the SO_2(g) + O_2 (g) or SO_2(g) + H_2O (g) poisoning of mesoporous Al_2O_3 and Pd/Al_2O_3 powder catalysts[6,7,22–27] revealed vibrational features due to i) sulfates on Pd[7,22] (1098, 1240, and 1460 cm^{-1}), ii) surface sulfates on Al_2O_3[6,7,22,25,26] (1374 and 1359 cm^{-1}), and iii) bulk-like sulfates on Al_2O_3 (i.e. $Al_2(SO_4)_3$)[7,22,23,26,27] (1189, 1264, 1276, 1328, and 1344 cm^{-1}).

5. Far-field Attenuated Total Reflectance (ATR)-IR Spectroscopy Measurements:

Figure S4 shows the background far field ATR-IR spectrum of the clean Pd(nanodisk)/Al_2O_3 model catalyst surface which was used to obtain the ATR-IR spectrum of the sulfur poisoned model catalyst surface shown in Figure 3j of the main text. To obtain the background ATR-IR spectrum clean Pd(nanodisk)/Al_2O_3 model catalyst surface given in Figure 3j, firstly, a background ATR-IR spectrum was initially acquired from a clean Si(100) substrate (i.e. without Pd nanodisks or Al_2O_3 thin film) and then, this spectrum was subsequently subtracted from the ATR-IR spectrum of the clean Pd(nanodisk)/Al_2O_3 model catalyst surface. ATR-IR spectrum of the clean Pd(nanodisk)/Al_2O_3 model catalyst surface shows vibrational features around 1108 and 1300 cm^{-1} which can readily be ascribed to transverse (TO) and longitudinal (LO) optical phonon absorptions of the SiO_x thin film which was formed on the Si(100) substrate during the lithographic model catalyst synthesis[8-9]. Note that the artifact (i.e. the dip) observed at 1108 cm^{-1} in Figure 3j originates from the poor spectroscopic compensation of these SiO_x features.

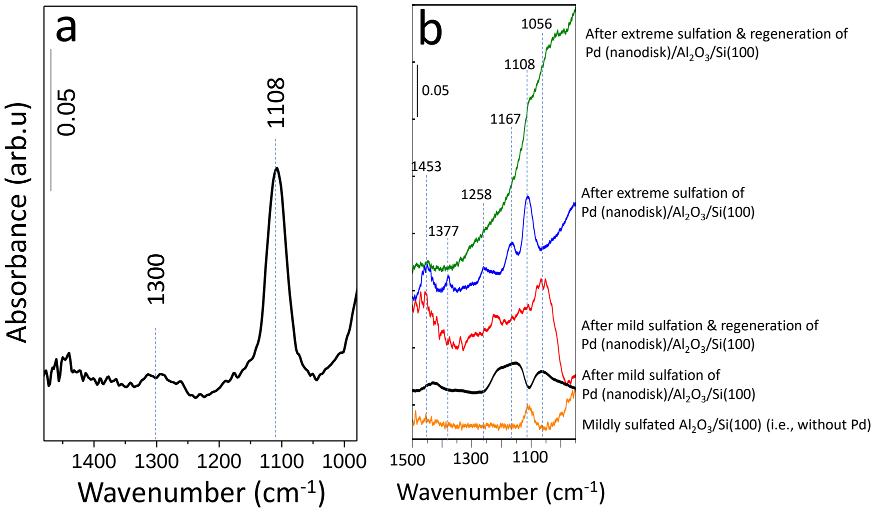

Figure S4. (a) Background ATR-FTIR spectrum of the clean Pd(nanodisk)/Al_2O_3 model catalyst surface. (b) ATR-FTIR analysis of the Al_2O_3/Si(100) model catalyst (without Pd) after mild sulfation, as well as Pd(nanodisk)/Al_2O_3 model catalyst after mild sulfation, after mild sulfation followed by regeneration, after extreme sulfation, and after extreme sulfation followed by regeneration. (Note that the black spectrum in (b) corresponding to the mildly poisoned Pd(nanodisk)/Al_2O_3 is the identical spectrum presented in Figure 3j of the main text but drawn with a different scale).

6. Atomic Force Microscopy (AFM) Height Profile Measurements:

Figure S5 illustrates the AFM height profile analysis of the clean Pd(nanodisk)/Al_2O_3 model catalyst as well as the Pd(nanodisk)/Al_2O_3 after mild sulfation, after mild sulfation followed by regeneration, after extreme sulfation, and after extreme sulfation followed by regeneration.

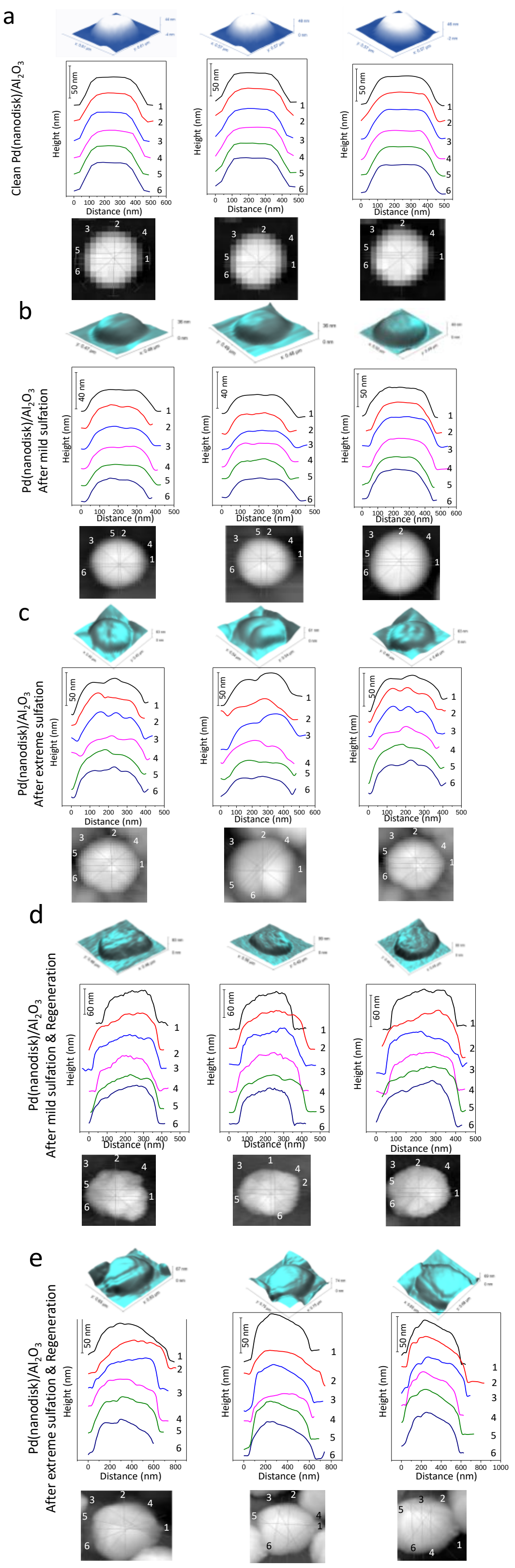

Figure S5. AFM height-profile analysis of clean Pd(nanodisk)/Al_2O_3 model catalyst surface, after mild sulfation, after mild sulfation and subsequent regeneration, after extreme sulfation, and after extreme sulfation and subsequent regeneration.

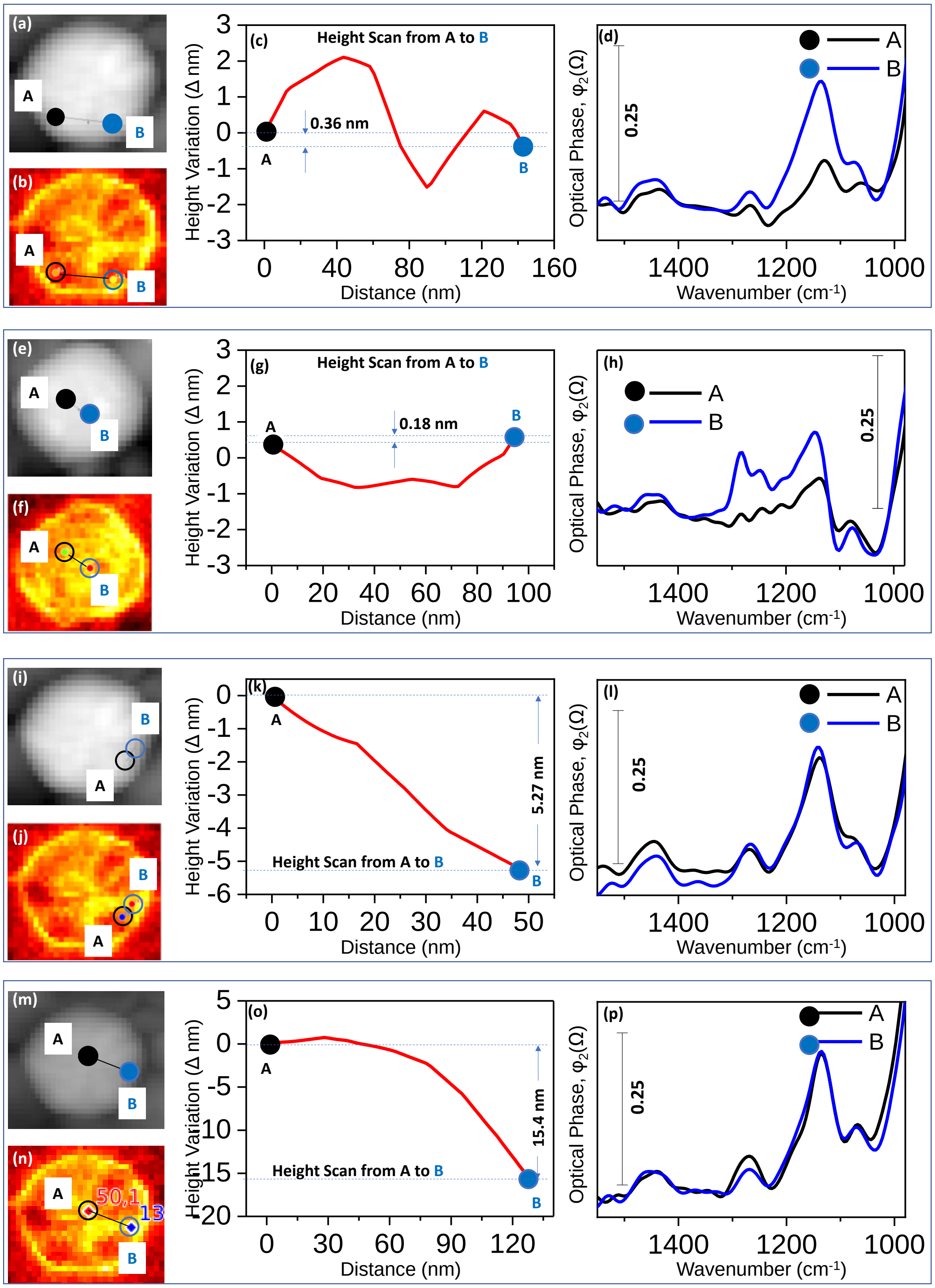

Figure S6. Control experiments demonstrating that observed nano-FTIR spectral intensities and line shapes in the current work are not correlated to the height-dependent variations in the near-field strength. First two panels on the top illustrate that nano-FTIR spectra obtained for two separate points on severely poisoned Pd(nanodisk)/Al_2O_3 model catalysts with identical relative heights reveal different spectral line shapes; while the last two panels depict that two separate points on Pd nanodisks with significant height difference yield identical nano-FTIR spectral line shapes. (a, e, i, m) AFM topographic images, (b, j, f, n) SNOM total IR reflection images, (c, g, k, o) height difference vs. distance curves, (d, h, l, p) nano-FTIR spectra.

References:

(1) Fredriksson, H.; Alaverdyan, Y.; Dmitriev, A.; Langhammer, C.; Sutherland, D. S.; Zäch, M.; Kasemo, B. Hole-Mask Colloidal Lithography. *Adv. Mater.* **2007**, *19* (23), 4297–4302.

(2) Wang, X.; Chen, J.; Zeng, J.; Wang, Q.; Li, Z.; Qin, R.; Wu, C.; Xie, Z.; Zheng, L. The Synergy between Atomically Dispersed Pd and Cerium Oxide for Enhanced Catalytic Properties. *Nanoscale* **2017**, *9*, 6643–6648.

(3) Kaden, W. E.; Wu, T.; Kunkel, W. A.; Anderson, S. L. Electronic Structure Controls Reactivity of Size-Selected Pd Clusters Adsorbed on TiO_2 Surfaces. *Science.* **2009**, *326* (5954), 826-829.

(4) Wu, T.; Kaden, W. E.; Kunkel, W. A.; Anderson, S. L. Size-Dependent Oxidation of Pdn ($n \leqslant 13$) on Alumina/NiAl(110): Correlation with Pd Core Level Binding Energies. *Surf. Sci.* **2009**, *603* (17), 2764–2770.

(5) Audi, A. A.; Sherwood, P. M. A. X-Ray Photoelectron Spectroscopic Studies of Sulfates and Bisulfates Interpreted by Xα and Band Structure Calculations. *Surf. Interface Anal.* **2000**, *29*, 265–275.

(6) Şentürk, G. S.; Vovk, E. I.; Zaikovskii, V. I.; Say, Z.; Soylu, A. M.; Bukhtiyarov, V. I.; Ozensoy, E. SO_x Uptake and Release Properties of TiO_2/Al_2O_3 and $BaO/TiO_2/Al_2O_3$ Mixed Oxide Systems as NOx Storage Materials. *Catal. Today* **2012**, *184* (1), 54–71.

(7) Wilburn, M. S.; Epling, W. S. Formation and Decomposition of Sulfite and Sulfate Species on Pt/Pd Catalysts: An SO2 Oxidation and Sulfur Exposure Study. *ACS Catal.* **2019**, *9* (1), 640–648.

(8) Wilburn, M. S.; Epling, W. S. A Summary of Sulfur Deactivation, Desorption, and Regeneration Characteristics of Mono- and Bimetallic Pd-Pt Methane Oxidation Catalysts: Pd:Pt Mole Ratio and Particle Size Dependency. *Emiss. Control Sci. Technol.* **2018**, *4*, 78–89.

(9) Sadokhina, N.; Smedler, G.; Nylén, U.; Olofsson, M.; Olsson, L. Deceleration of SO_2 Poisoning on $PtPd/Al_2O_3$ Catalyst during Complete Methane Oxidation. *Appl. Catal. B Environ.* **2018**, *236*, 384–395

(10) Zhang, I. Y.; Zwaschka, G.; Wang, Z.; Wolf, M.; Campen, R. K.; Tong, Y. Resolving the Chemical Identity of H_2SO_4 Derived Anions on Pt(111) Electrodes: They're Sulfate. *Phys. Chem. Chem. Phys.* **2019**, *21*, 19147–19152.

(11) Wu, C. H.; Pascal, T. A.; Baskin, A.; Wang, H.; Fang, H. T.; Liu, Y. S.; Lu, Y. H.; Guo, J.; Prendergast, D.; Salmeron, M. B. Molecular-Scale Structure of Electrode-Electrolyte Interfaces: The Case of Platinum in Aqueous Sulfuric Acid. *J. Am. Chem. Soc.* **2018**, *140* (47), 16237–16244.

(12) Hoshi, N.; Kuroda, M.; Koga, O.; Hori, Y. Infrared Reflection Absorption Spectroscopy of the Sulfuric Acid Anion on Low and High Index Planes of Palladium. *J. Phys. Chem. B* **2002**, *106* (35), 9107–9113.

(13) Faguy, P. W.; Markovic, N.; Adzic, R. R.; Fierro, C. A.; Yeager, E. B. A Study of Bisulfate Adsorption on Pt(111) Single Crystal Electrodes Using in Situ Fourier Transform Infrared Spectroscopy. *J. Electroanal. Chem.* **1990**, 289, 245-262.

(14) Sawatari, Y.; Inukai, J.; Ito, M. The Structure of Bisulfate and Perchlorate on a Pt(111) Electrode Surface Studied by Infrared Spectroscopy and Ab-Initio Molecular Orbital Calculation. *J. Electron Spectros. Relat. Phenomena* **1993**, *64–65* (C), 515–522.

(15) Nart, F. C.; Iwasita, T.; Weber, M. Vibrational Spectroscopy of Adsorbed Sulfate on Pt(111). *Electrochim. Acta* **1994**, *39* (7), 961–968.

(16) Shingaya, Y.; Ito, M. Interconversion of a Bisulfate Anion into a Sulfuric Acid Molecule on a Pt(111) Electrode in a 0.5 M H_2SO_4 Solution. *Chem. Phys. Lett.* **1996**, *256* (4–5), 438–444.

(17) Faguy, P. W.; Marinković, N. S.; Adžić, R. R. An in Situ Infrared Study on the Effect of PH on Anion Adsorption at Pt(III) Electrodes from Acid Sulfate Solutions. *Langmuir* **1996**, *12* (2), 243–247.

(18) Thomas, S.; Sung, Y. E.; Kim, H. S.; Wieckowski, A. Specific Adsorption of a Bisulfate Anion on a Pt(111) Electrode. Ultrahigh Vacuum Spectroscopic and Cyclic Voltammetric Study. *J. Phys. Chem.* **1996**, *100* (28), 11726–11735.

(19) Hoshi, N.; Sakurada, A.; Nakamura, S.; Teruya, S.; Koga, O.; Hori, Y. Infrared Reflection Absorption Spectroscopy of Sulfuric Acid Anion Adsorbed on Stepped Surfaces of Platinum Single-Crystal Electrodes. *J. Phys. Chem. B* **2002**, *106* (8), 1985–1990.

(20) Hoshi, N.; Kuroda, M.; Ogawa, T.; Koga, O.; Hori, Y. Infrared Reflection Absorption Spectroscopy of the Sulfuric Acid Anion Adsorbed on Pd(S)-[n(111) × (111)] Electrodes. *Langmuir* **2004**, *20* (12), 5066–5070.

(21) Liu, S.; Wu, X.; Weng, D.; Li, M.; Fan, J. Applied Catalysis B : Environmental Sulfation of Pt/Al_2O_3 Catalyst for Soot Oxidation : High Utilization of NO_2 and Oxidation of Surface Oxygenated Complexes. *"Applied Catal. B, Environ.* **2013**, *138–139* (2), 199–211.

(22) Mowery, D. L.; McCormick, R. L. Deactivation of Alumina Supported and Unsupported PdO Methane Oxidation Catalyst: The Effect of Water on Sulfate Poisoning. *Appl. Catal. B Environ.* **2001**, *34* (4), 287–297.

(23) Chang, C. C. Infrared Studies of SO_2 on γ-Alumina. *J. Catal.* **1978**, *53* (3), 374–385.

(24) Wu, Q.; Gao, H.; He, H. Conformational Analysis of Sulfate Species on Ag/Al_2O_3 by Means of Theoretical and Experimental Vibration Spectra. *J. Phys. Chem. B* **2006**, *110* (16), 8320–8324.

(25) B. Mitchell, M.; N. Sheinker, V.; G. White, M. Adsorption and Reaction of Sulfur Dioxide on Alumina and Sodium-

Impregnated Alumina. *J. Phys. Chem.* **1996**, *100* (18), 7550–7557.
(26) Waqif, M.; Saur, O.; c. Lavalley, J.; Perathoner, S.; Centi, G. Nature and Mechanism of Formation of Sulfate Species on Copper/Alumina Sorbent-Catalysts for Sulfur Dioxide Removal. *J. Phys. Chem.* **2002**, *95* (10), 4051–4058.
(27) Bounechada, D.; Fouladvand, S.; Kylhammar, L.; Pingel, T.; Olsson, E.; Skoglundh, M.; Gustafson, J.; Di Michiel, M.; Newton, M. A.; Carlsson, P.-A. Mechanisms behind Sulfur Promoted Oxidation of Methane. *Phys. Chem. Chem. Phys.* **2013**, *15* (22), 8648–8661.
